# Effects of Illness Management and Recovery: A Multicenter Randomized Controlled Trial

**DOI:** 10.3389/fpsyt.2021.723435

**Published:** 2021-12-14

**Authors:** Bert-Jan Roosenschoon, Jaap van Weeghel, Mathijs L. Deen, Emmie W. van Esveld, Astrid M. Kamperman, Cornelis L. Mulder

**Affiliations:** ^1^Department of Psychiatry, Epidemiological and Social Psychiatric Research Institute, Erasmus MC University Medical Centre, Rotterdam, Netherlands; ^2^Parnassia Academy, Parnassia Psychiatric Institute, Den Haag, Netherlands; ^3^Department of TRANZO, Tilburg School of Social and Behavioral Sciences, Tilburg University, Tilburg, Netherlands; ^4^Faculty of Social and Behavioral Sciences, Institute of Psychology, Leiden University, Leiden, Netherlands; ^5^Yulius Mental Health, Dordrecht, Netherlands; ^6^Antes Mental Health Care, Parnassia Psychiatric Institute, Rotterdam, Netherlands

**Keywords:** illness management and recovery (IMR), psychosocial treatment, recovery, schizophrenia, self-management, severe mental illness, fidelity, completion

## Abstract

There have been inconsistent findings in the literature with respect to the efficacy of Illness Management and Recovery (IMR) in the psychosocial treatment of people with schizophrenia or other severe mental illnesses. This study aimed to comprehensively investigate the effectiveness of IMR, including the impact of completion and fidelity. In this randomized controlled trial (RCT), 187 outpatients received either IMR plus care as usual (CAU) or only CAU. Multilevel modeling was implemented to investigate group differences over an 18-month period, comprising 12 months of treatment and six months of follow-up. The primary outcome was overall illness management, which was assessed using the client version of the IMR scale. Secondary outcomes included measures regarding illness management, clinical, personal, and functional recovery, and hospitalizations. The interviewers were blinded to group allocation. This clinical trial was registered with the Netherlands Trial Register (NL4931, NTR5033). Patients who received IMR showed statistically significant improvement in self-reported overall illness management (the primary outcome). Moreover, they showed an improvement in self-esteem, which is a component of personal recovery. There were no effects within the other questionnaires. There were also no statistically significant between-group differences in terms of hospitalizations. Patients in both groups showed statistically significant improvement in clinician-rated overall illness management, social support, clinical and functional recovery, and self-stigma over time. IMR completion was associated with stronger effects. High IMR fidelity was associated with self-esteem. This study confirms the efficacy of IMR in overall illness self-management. To our knowledge, this is the first RCT on IMR to explore the impact of fidelity on treatment efficacy. Future studies should further establish efficacy in personal recovery. To improve efficacy, it appears important to promote IMR completion and fidelity.

## Introduction

Patients with schizophrenia and other severe mental illnesses (SMIs) face major challenges in achieving personal goals and fully participating in society. This is due to their recurring symptomatology, cognitive impairment, loss of social support, and societal barriers such as stigma (1, 2). Although treatment with psychopharmacological drugs facilitates reductions in symptom severity and relapse, there is a need for effective psychosocial interventions to support participants in illness self-management. The aim of these interventions is to develop fulfilling and valued workplace roles and social connections, to obtain housing, and to facilitate self-determination and well-being (1, 3–6). Illness management programs, including Illness Management and Recovery (IMR), have been developed to support individuals with SMIs in addressing the physical, social, and emotional impact of their persistent condition. These programs seek to improve the course of illness (1, 7, 8).

**Figure 1:**
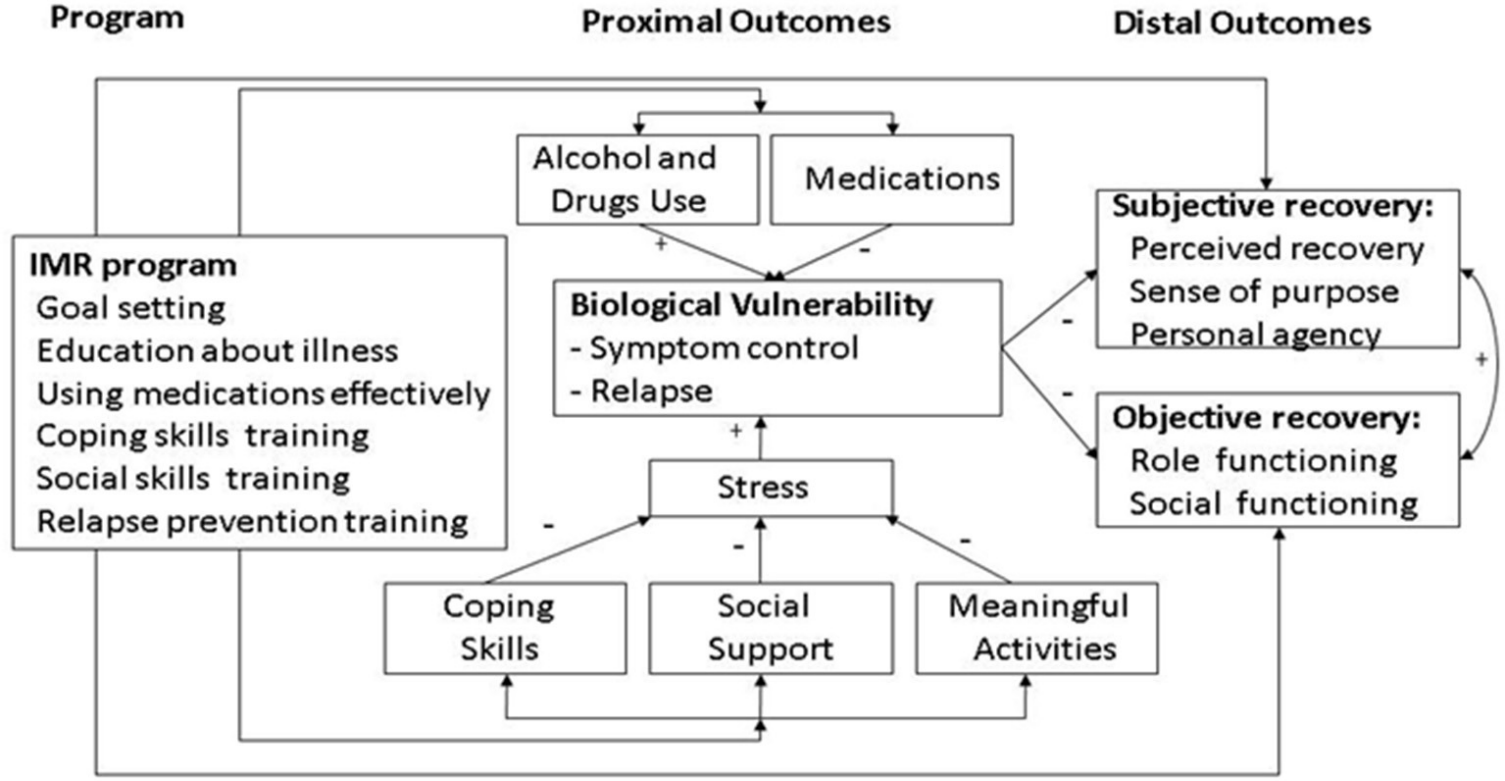
Conceptual framework of illness management and recovery (3).

IMR is a structured psychosocial program that promotes illness self-management in people with schizophrenia and other SMIs. It was developed based on an empirical literature review with regard to teaching illness self-management strategies (9). IMR combines psychoeducation, behavioral tailoring for medication adherence, relapse-prevention training, and cognitive-behavioral training in social and coping skills (3). The various individual components of the IMR program are not new; however, the novelty of IMR results from offering these components as an integrated, introductory package (6). The working mechanisms underlying IMR have been suggested in its previously published conceptual framework (Figure 1), indicating that progress toward recovery may be achieved by combining better illness management with the pursuit of personal goals (3). Recovery is a multidimensional concept comprised of three subtypes that are not mutually exclusive (10–14): clinical or symptomatic recovery (4, 15); functional or objective recovery (3), which is defined as the degree of vocational and social functioning (14, 16–18), and personal or subjective recovery (3, 19), whose key elements are summarized via the acronym CHIME: connectedness, hope, identity, meaning in life, and empowerment (20). IMR is currently implemented in various countries, including the US, the Netherlands, Denmark, Norway, Sweden, Spain, Japan, and Singapore.

A recent systematic review and meta-analysis of 37 studies on self-management interventions found that these interventions reduced symptoms and the length of hospital stay, as well as enhancing functioning and quality of life. Moreover, there were statistically significant positive effects on subjective measures of recovery, self-rated recovery, and self-efficacy (7). Although this review indicated the relevance of self-management interventions, it only included nine (24%) studies on IMR. Therefore, there is a need for more IMR-specific experimental evidence.

In September 2018, we conducted a literature search that yielded 65 studies on IMR, including six randomized controlled trials (RCTs) (21–28). These six RCTs yielded inconsistent results. Regarding our primary outcome—illness self-management measured using the client version of the IMR scale—three RCTs reported positive results for IMR as compared with the control group (22, 23, 28), while the remaining three RCTs reported null results (21, 24, 25, 27). Regarding illness management measured using the clinician version of the IMR scale, four RCTs reported positive results for IMR (21–23, 28), while one other RCT reported null results (25, 27). Three RCTs reported that IMR had positive effects on reducing psychiatric symptoms (22, 23, 28), while the other RCTs identified in the literature search reported null results (22, 24, 26, 27). Regarding hospitalizations, only one RCT reported positive results (28); the remaining RCTs reported null results (21–24, 26). Three RCTs reported no effect on personal recovery (23–25, 27) and one RCT reported no effect on employment (22). Two RCTs did not observe any effects of IMR (24–26).

Differences in results between these studies may be related to variations in patient populations, sample sizes, control group characteristics, the duration of IMR, drop-out rates, and model fidelity (6, 29). The present study is focused on two implementation aspects that may have contributed to the inconsistency in the results of earlier RCTs: model fidelity and IMR completion. Fidelity is defined as the degree of adherence to the standards and principles of a program model (30, 31). There are indications that higher fidelity to empirically supported mental health program models, notably evidence-based practices (EBPs), is predictive of better client outcomes for people with SMIs (32). With respect to this predictive validity, there is some evidence regarding several interventions for this target group: Assertive Community Treatment (ACT) (33–37), Individual Placement and Support (IPS) (38), and IMR (39).

Before conducting the current study, we conducted a pilot study to explore the feasibility of a randomized controlled trial. This pilot study indicated that an RCT appeared feasible. However, the results likewise indicated a 50% dropout rate from the treatment regimen (30). The relevance of IMR completion (22, 40), including with respect to both fidelity and completion (27), has been suggested in prior research. Therefore, the current study explored the impact of fidelity and IMR completion rate on the efficacy of this intervention. The study design was customized to facilitate completion analysis: *a priori*, we chose to assign more clients to the experimental condition (IMR) than to the CAU group (6).

Although IMR appeared promising for implementation in the Netherlands, the inconsistent findings within previous studies indicated the need for further thorough research on IMR efficacy and outcomes. Therefore, the present study aimed to compare IMR plus care as usual (CAU) with CAU alone in patients with schizophrenia or other SMIs. Using multiple outcome measures of illness self-management, illness outcomes, and recovery, the present study sought to thoroughly assess the effects of IMR. We formulated multiple hypotheses as follows: (1) IMR + CAU allows for better illness management as well as reducing symptoms and relapses as compared with CAU alone; (2) IMR + CAU allows for better personal and functional recovery as compared with CAU alone; and (3) IMR+CAU-related improvement is associated with the fidelity of IMR implementation (6). The adaptation of the study design to facilitate completion analysis was based on the proposition that a higher rate of completion is associated with stronger effects.

## Methods

### Study Design

This multicenter, parallel-group, single-blinded RCT was conducted at two mental health care institutions in the Netherlands (6). Ethical approval was obtained at the Erasmus University Medical Center (METC registration number NL38605.078.12). The inclusion criteria were as follows: age 18–65 years; diagnosis with an SMI such as schizophrenia or a persistent mood disorder with or without comorbid disorders (i.e., substance abuse and personality disorders); and receiving outpatient treatment in the greater Rotterdam area. Exclusion criteria comprised previous participation in IMR training and insufficient Dutch language skills. Participants were recruited through clinician referrals from 14 participating community mental health teams.

### Measures

The *primary outcome measure* was the IMR scale (client version) (6). Both the client and clinician versions of the IMR scale allow for the overall measurement of illness self-management, and both scales are composite measures of various self-management components. The 15 scale items comprise the key elements of IMR training, including progress toward goals, knowledge regarding mental illness, relapse-prevention planning, involvement with significant others, coping with symptoms, medication adherence, substance abuse, and symptom distress (3, 41–43).

Moreover, we sought to determine whether individually assessing the scale components had any effect on study results and interpretation. Therefore, in addition to the clinician version of the IMR scale and measuring instruments with respect to clinical, functional, and personal recovery, we included five separate scales regarding components of illness self-management as secondary outcome measures in the current study (6).

*Secondary outcome measures* included the IMR Scale (clinician version) (43) and five scales assessing specific aspects of illness management as follows: coping (Coping Self-Efficacy Scale) (44), social support (Multidimensional Scale of Perceived Social Support) (45), medication adherence (treatment-adherence-subscale of the clinician-rated Service Engagement Scale) (46), problems with alcohol or drugs (item 24 of the Addiction Severity Index) (47), and psychiatric insight (Insight Scale) (48). Clinical recovery was analyzed using the Brief Symptom Inventory (49). Hospitalizations were assessed and operationalized as the occurrence of hospitalizations and the lengths of hospital stay. Functional recovery was assessed using the Social Functioning Scale (50). Personal recovery was assessed using the Mental-Health Recovery Measure (MHRM), a composite personal recovery scale that measures “self-empowerment,” “learning and new potentials,” and “spirituality” (51), the Internal Stigma of Mental Illness scale (ISMI) (52), and the Self-Esteem Rating Scale-Short Form (SERS-SF) (6, 53). The IMR Fidelity Scale was used to measure fidelity to IMR program implementation at the group level (31, 54). Details regarding all scales have been described previously (6, 55).

### Procedures

During interviews with an assistant researcher, the caseloads of all clinicians on the community mental health teams were reviewed by the clinicians themselves for their potential suitability for IMR. The clinicians then asked the selected clients about participating in IMR and about their willingness to be informed about the study. If clients expressed interest and agreed to be contacted by an assistant researcher with more detailed information, the assistant researcher explained the study objectives, the randomization procedure, and the three measurement moments. The clients were then asked if they were willing to participate in the study. After written informed consent was given, the baseline interview was followed by randomization (6). For allocation of participants to receive IMR plus CAU (i.e., the intervention group) or CAU only (i.e., the control group), we used computer-generated random permuted blocks of five stratified across the treatment teams. Blocks were independently constructed by a research assistant using a randomization plan (http://www.randomization.com). The results of our pilot study suggested an expected 50% dropout rate from treatment within the experimental condition (55). Therefore, to facilitate completer analyses, we allocated more participants to the intervention group than to the control group with a ratio of 3:2. Written informed consent was obtained from all participants prior to participation.

Since research assistants were blinded to group allocation, the data collection procedure was single-blinded (11). Before each assessment, participants and clinicians were instructed through letter or e-mail not to disclose the treatment assignment. At the start of each session, the participants were verbally reminded of this requirement of the investigation.

Assessments were performed at baseline (T1), after 12 months (post-treatment, T2), and after 18 months (six months of follow-up, T3). The research assistants, who were centrally instructed, met the study participants at several institute branches to conduct the assessments.

The principal researcher and one of the two co-auditors assessed IMR fidelity in 15 IMR groups within one-day site visits near the end of the treatment course. Both assessors performed independent scoring following a standard procedure (54). In the case of differences or disagreements, the assessors established a consensus score. The principal investigator was trained on how to use the IMR fidelity scale by two American specialists (6). The two trained co-auditors were a psychologist and an advanced nurse practitioner. In addition to semi-structured interviews with participants and trainers, assessments involved one observation session and checking forms, such as anonymized IMR Goal Tracking Sheets (6). All IMR elements were reviewed with all respondents during the interviews. This included questioning about elements that were not used during the observed session. Thus, we were able to obtain a good overall picture of the fidelity within each group and we were able to assess all 13 items of the IMR fidelity scale. Periodically, the researchers provided feedback regarding the assessment results in the supervision groups.

To assess the impact of fidelity, patients who received IMR were assigned the fidelity score of their group. This score indicates the fidelity level of the IMR model as applied to the participant.

### IMR Content and Services

All study participants received CAU comprising standard outpatient psychiatric care (including case management, multidisciplinary psychiatric and psychological treatment, and rehabilitation services). The treatment contacts for individual case management involved face-to-face meetings with a mental health nurse, a social worker, or another psychiatric counselor at two-week intervals. Additionally, a psychiatrist was consulted at least once a year as appropriate. Typical contacts with case managers involved individualized recovery support and structuring conversations on all life domains.

In the intervention group, these usual services were provided together with IMR, a comprehensive structured training program for people with SMI. IMR was provided in a group format in weekly 90-min sessions. In accordance with the IMR fidelity scale, these groups included up to eight people. However, the exact group size varied per group and per session. The IMR intervention comprised eleven modules as well as practitioner guides and handouts for the participants. The eleven modules included Recovery Strategies, Basic Facts about Mental Illness, the Stress-Vulnerability Model, Building Social Support, Using Medication Effectively, Drug and Alcohol Use, Reducing Relapses, Coping with Stress, Coping with Persistent Symptoms, Getting Your Needs Met in the Mental-Health System, and Healthy Lifestyles (56). The original American text was translated into Dutch and was adapted to the Dutch context as appropriate. Each group of patients who received IMR was guided by two trainers, who used a combination of motivation-enhancement strategies (including conveying confidence and exploring the pros and cons of change), educational strategies (including interactive teaching, breaking down information, and checking for understanding), and cognitive-behavioral techniques (including shaping, modeling, and role-playing) (3). Peer-group support, coping, and social skills training are key IMR elements. Workbooks and homework assignments were accessible through an e-health module (6). During the first module—Recovery Strategies—the participants identified their personal goals during the program. For half of each session, some participants worked toward these goals. During the other half of each session, all participants worked on the module subjects with the help of the handouts. Our pilot study demonstrated that completing the modules required an average of three to four sessions (55).

IMR was mostly provided by mental health nurses or social workers, recruited from among the group of case managers in the CMHC teams who were interested in providing the IMR intervention. All case managers were given the opportunity to provide IMR. Most case managers had years of professional experience and had previously been trained in psychiatric rehabilitation methodologies, including the Boston University Approach to Psychiatric Rehabilitation (57). As part of the institutes' regular quality care program, all IMR trainers underwent two days of training in IMR and underwent additional four-hour training sessions twice annually. The IMR trainers underwent two hours of group supervision by a senior counselor at two-week intervals.

### Statistical Analysis

Based on the effect sizes observed in the three RCTs on IMR published at the time of designing the current study (21–23), we anticipated a moderate effect size of 0.40 with respect to the primary outcome variable (i.e., the self-rated IMR scale) (41, 42). Based on power analyses with three measurement times (mixed models), equal allocation to the experimental and control groups, a power of 0.80, alpha set at 0.05, and an effect size of 0.40, we determined that it would be necessary to randomize 148 clients: 74 to the experimental condition and 74 to the CAU control group (6, 58, 59). Due to the planned 3:2 randomization we aimed for 111 participants (74 × 3:2) in the experimental condition.

Linear mixed models (LMM) were implemented to investigate between-group differences (IMR vs. CAU) over an 18-month period, comprising 12 months of treatment and six months of follow-up (59). To assess treatment effects, we adopted the following analysis protocol for all outcomes (6). We first determined the best-fitting model using a stepwise modeling procedure. Subsequently, time and conditions were entered into the model; a time × condition interaction term was included only if the inclusion of this term improved the model based on the Akaike Information Criterion. A statistically significant time × condition interaction was considered to represent a treatment effect. For all outcomes, the estimated marginal means and 95% confidence intervals (CI) were calculated for the three measurement moments. To assess between-group effect sizes, we calculated Cohen's *d*s (0.2–0.3, small; 0.5, medium; >0.8, large) (60). As LMMs use the full data set, this methodology retains people with missing values; thus, imputation is not necessarily beneficial in this context (61).

For all outcomes, we followed the same procedure to examine the robustness of our findings within a sensitivity analysis among IMR completers. A “completer” was defined as a patient who had attended ≥50% of all scheduled sessions. To explore the possibility of selective IMR non-completion, the patients' attendance was analyzed via logistic regression using 12 variables representing baseline characteristics.

Hospitalization was analyzed in two ways. First, to examine between-group differences in the odds of hospitalization during the year following T2, we performed logistic regression analysis with respect to hospitalization (dichotomized), with T2 as the dependent variable, the treatment group as the independent variable, and hospitalization (dichotomized) one year prior to T1 as a confounder. Second, we determined difference scores with respect to the length of stay (days) at one year before and after treatment. Between-group differences were tested using the Mann–Whitney test.

In our study protocol, we hypothesized that IMR-related improvement would be associated with the fidelity of IMR implementation (6). Therefore, we explored the impact of fidelity on outcomes. This analysis included all participants with fidelity scores who had attended at least ten IMR sessions, indicating consistent engagement with the IMR program. Participants were divided into high and moderate-to-low fidelity groups, with mixed models used to assess differences in time effects between the control group and the two experimental subgroups. The control group was considered the reference group. The model components were comprised of the three measurement moments, the three subgroups (high fidelity, moderate/low fidelity, and control), and their interactions. In cases where there was an overall statistically significant interaction effect, we interpreted the parameter estimates for these interaction effects as appropriate. The cutoff scores for high, moderate, and low fidelity within the IMR fidelity scale were ≥4.0, 3.0–4.0, and <3.0, respectively (54).

Statistical analyses were performed using the Statistical Package for the Social Sciences (version 25.0; SPPS, Inc., Chicago, IL, USA). Multiplicity adjustments, including Bonferroni and Benjamini–Hochberg corrections (62), are often applied. However, these methodologies have substantial limitations (63, 64). This study applied 11 secondary outcomes, selected carefully *a priori*, in order to explore the effects of various components of the conceptual framework for the IMR program (3). Our analyses followed a pre-specified protocol (6) and we present a qualitative interpretation of our findings. Additionally, as a sensitivity analysis, we present the impact of Benjamini–Hochberg corrections on our results.

Due to an administrative oversight, the prospective trial registration was initially overlooked; however, this omission was noticed halfway through the data collection period and the trial was registered with the Netherlands Trial Register (NL4931, NTR5033). Details of our initial ethical approval and protocol are provided in the Supplementary Material.

## Results

**Figure 2:**
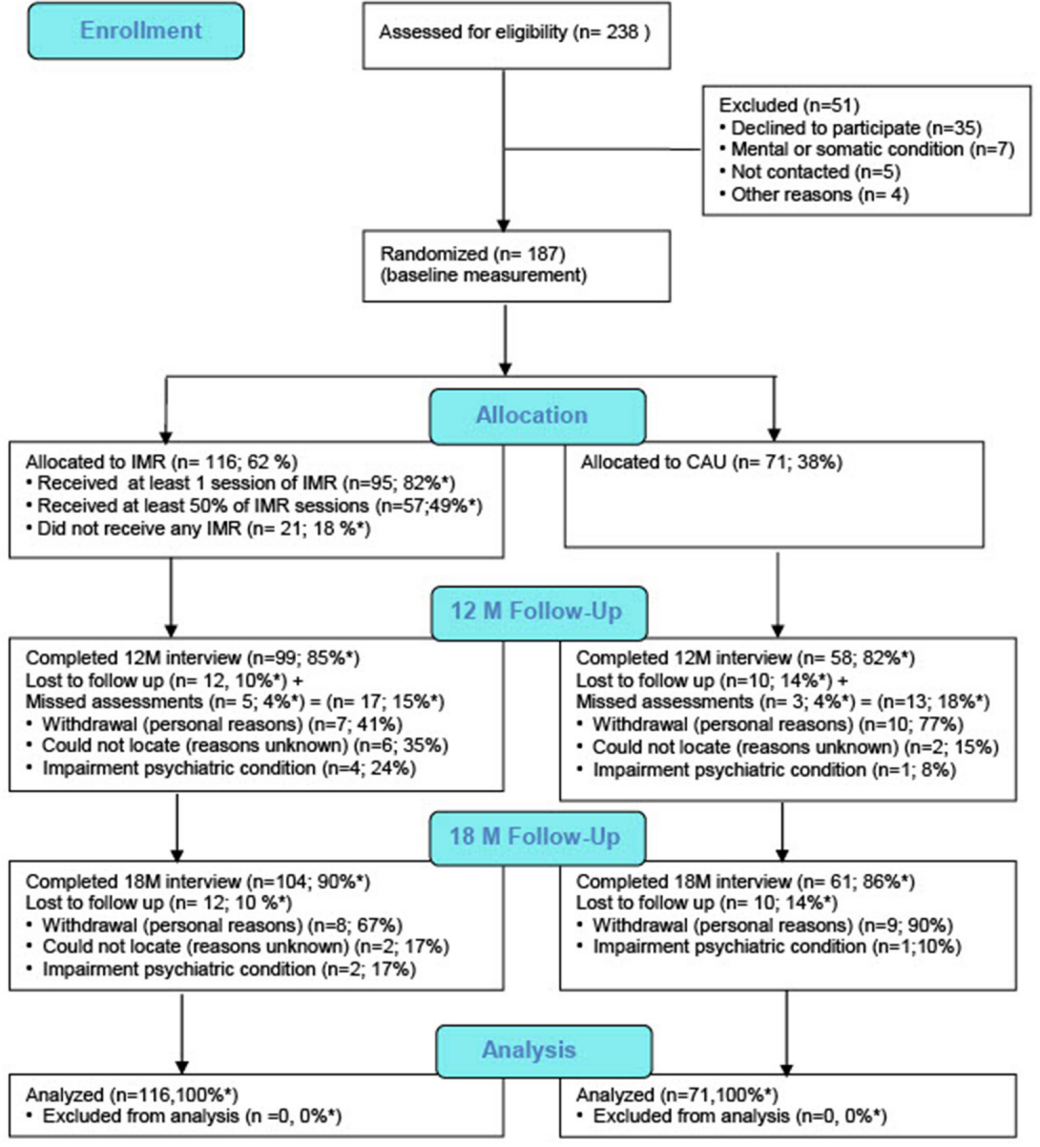
CONSORT Flowchart. *Percentage of participants allocated to condition.

Between October 2012 and May 2014, we randomly assigned 187 participants (3:2 ratio) to receive IMR + CAU (*n* = 116) or CAU alone (*n* = 71; Figure 2 shows the CONSORT flow chart for study enrollment). There were no between-group differences in baseline demographic or medical characteristics (Table 1), and only 9.4% missing data.

**Table 1:** Demographic and clinical characteristics of study participants at baseline.

	**Control**	**IMR**		**Control**	**IMR**
	**Group (*n* = 71)**	**Group (*n* = 116)**		**Group (*n* = 71)**	**Group (*n* = 116)**
Age (years)	43.6 (10.7)	44.7 (10.2)	Start of problems		
Sex			≤10 years previous to IMR	28 (39%)	35 (30%)
Male	40 (56%)	59 (51%)	>10 years previous to IMR	43 (61%)	81 (70%)
Female	31 (44%)	57 (49%)	Number of admissions		
Living situation			None	20 (28%)	28 (24%)
Living alone	49 (69%)	62 (53%)	1–2	25 (35%)	44 (38%)
Living with others	16 (22%)	32 (28%)	≥3	26 (37%)	44 (38%)
Living in institution^*^	6 (9%)	22 (19%)	Length hospitalization		
Education level			Not hospitalized	20 (28%)	28 (24%)
Low	27 (38%)	42 (36%)	≤1 year	41 (58%)	53 (46%)
Middle	33 (46%)	46 (40%)	>1 year	10 (14%)	35 (30%)
High	11 (16%)	28 (24%)			
Native country			IMRS		
Netherlands	46 (65%)	90 (78%)	IMRS client version	3.3 (0.5)	3.3 (0.5)
Western-immigrant	9 (13%)	7 (6%)	IMRS clinician version	3.2 (0.4)	3.3 (0.4)
Non-western immigrant	16 (22%)	19 (16%)	IM constituents		
Source of income			MSPSS	4.7 (1.5)	5.0 (1.5)
Employment	5 (7%)	7 (6%)	CSES	130.8 (51.0)	137.2 (50.6)
Unemployment-, sickness-, or invalidity benefit	49 (70%)	77 (66%)	SES	10.8 (1.9)	10.8 (1.9)
			IS	9.4 (3.4)	9.8 (2.6)
Social security benefit	15 (21%)	26 (23%)	ASI	0.5 (1.1)	0.5 (1.3)
No income	1 (1%)	5 (4%)	Recovery-scales		
Missing	1 (1%)	1 (1%)	SFS	104.9 (8.4)	106.0 (8.9)
Diagnosis^**^			BSI	1.32 (0.9)	1.23 (0.9)
Psychotic disorders Y/N	35 (49%)	71 (61%)	MHRM	69.0 (19.04)	70.2 (21.0)
Mood disorders Y/N	26 (37%)	35 (30%)	SERS-SF	6.9 (24.0)	11.5 (23.6)
Personality disorders Y/N	25 (28%)	33 (31%)	ISMI	2.2 (0.5)	2.1 (0.5)

*Data are Mean (SD) or n (%). Y/N, Yes/No. IMRS, Illness Management and Recovery scale; MSPSS, Multidimensional*.

*Scale of Perceived Social Support. CSES, Coping Self Efficacy Scale; SES, Service Engagement Scale treatment adherence subscale*.

*IS, Insight Scale; ASI, Addiction Severity Index, item 24; BSI, Brief Symptom Inventory; SFS, Social Functioning Scale; MHRM, Mental Health Recovery Measure; SERS-SF, Self-Esteem Rating Scale-Short Form; ISMI, Internal Stigma of Mental Illness*.

* *Sheltered living or in hospital*.

** *One person can have had more than one diagnosis*.

On average, participants in the experimental group (*n* = 116) attended 23.57 IMR sessions [standard deviation (SD) = 21.09], representing 44.6% of all scheduled sessions (SD = 37.3%). IMR completers (*n* = 57, 49%) attended an average of 42.4 sessions (SD = 12.64), representing 80.1% of the total number of scheduled sessions (SD = 13.05%).

### Primary Outcome

As compared with the control group, the IMR group showed a statistically significant improvement in the *primary outcome measure* (illness self-management measured with the client version of the IMR scale; *p* = 0.048) (Table 2). The largest improvement, though still with a small effect size, was shown at follow-up (Table 3).

**Table 2:** Fixed effects of IMR on all outcomes from final linear mixed model, in the Intention to treat sample.

**Illness Management and Recovery Scales**				**Illness Management constituents**				**Clinical, Functional and Personal Recovery**			
	**B**	**95% CI**	***p***		**B**	**95% CI**	***p***		**B**	**95% CI**	***p***
**IMRS client version^a^**				**Social Support (MSPSS)**				**Clinical Recovery (BSI)**			
Intercept	3.30	3.18 to 3.41	<0.001	Intercept	4.68	4.35 to 5.00	<0.001	Intercept	1.26	1.14 to 1.38	<0.001
Time	0.04	−0.01 to 0.10	0.15	Time	0.11	−0.01 to 0.20	0.02	Time	−0.05	−0.09 to −0.004	0.03
Condition	0.02	−0.13 to 0.16	0.82	Condition	0.35	−0.06 to 0.75	0.09	Condition^b^			
Condition × time	0.07	0.0007 to 0.15	0.048^*^	Condition × time^b^				Condition × time^b^			
**IMRS clinician version**				**Coping self efficacy (CSES)**				**Functional Recovery (SFS)**			
Intercept	3.21	3.12 to 3.30	<0.001	Intercept	130.28	118.95 to 141.62	<0.001	Intercept	105.37	104.12 to 106.62	<0.001
Time	0.08	0.02 to 0.15	0.007	Time	2.59	−2.26 to 7.43	0.29	Time	0.83	0.35 to 1.31	0.001
Condition	0.08	−0.05 to 0.18	0.28	Condition	7.67	−6.72 to 22.05	0.29	Condition^b^			
Condition × time	0.08	−0.02 to 0.13	0.18	Condition × time	4.13	−1.98 to 10.25	0.18	Condition × time^b^			
				**Medication adherence (SES)**				**Pers. Recovery (MHRM)**			
				Intercept	10.75	10.54 to 10.97	<0.001	Intercept	68.68	64.02 to 73.34	<0.001
				Time^b^				Time	−0.22	−2.25 to 1.81	0.83
				Condition^b^				Condition	1.45	−4.47 to 7.37	0.63
				Condition × time^b^				Condition × time	2.12	−0.44 to 4.68	0.10
				**Insight (IS)**				**Pers. Recovery (SERS-SF)**			
				Intercept	9.64	9.30 to 9.99	<0.001	Intercept	6.62	1.27 to 11.98	0.02
				Time^b^				Time	−0.64	−2.93 to 1.64	0.58
				Condition^b^				Condition	4.90	−1.90 to 11.7	0.16
				Condition × time^b^				Condition × time	3.71	0.84 to 6.59	0.01^*^
				**Addiction (ASI)**				**Pers. Recovery (ISMI)**			
				Intercept	0.39	0.27 to 0.52	<0.001	Intercept	2.19	2.08 to 2.30	<0.001
				Time^b^				Time	−0.04	−0.07 to −0.01	0.04
				Condition^b^				Condition	−0.11	−0.25 to 0.03	0.12
				Condition × time^b^				Condition × time^b^			

a *Primary Outcome Measure*.

b *These cells remain empty because, with stepwise modeling, “the best fitting model” was found at an earlier step*.

* *A statistically significant interaction effect, p < 0.05. IMRS, Illness Management and Recovery scale; MSPSS, Multidimensional Scale of Perceived Social Support; CSES, Coping Self- Efficacy Scale; SES, Service Engagement Scale, subscale treatment adherence; IS, Insight Scale; ASI,Addiction Severity Index, item 24; BSI, Brief Symptom Inventory; SFS, Social Functioning Scale; MHRM, Mental Health Recovery Measure; SERS-SF, Self-Esteem Rating Scale-Short Form; ISMI, Internal Stigma of Mental Illness*.

**Table 3:** Estimated outcomes as a function of IMR, estimated from linear mixed models in the Intention to treat sample.

	**Baseline**				**Post treatment (12 months)**				**Follow-up (18 months)**			
	***N***	**Mean (95% *CI*)**	**Mean difference (95% *CI*)**	***d***	***N***	**Mean (95% *CI*)**	**Mean difference (95% CI)**	***D***	***N***	**Mean (95% *CI*)**	**Mean difference (95% *CI*)**	***d***
**PRIMARY OUTCOME**												
**IMRS client version**												
Experimental	116	3.32 (3.22, 3.41)	0.03 (−0.11, 0.17)	0.06	99	3.43 (3.33, 3.53)	0.05 (−0.11, 0.22)	0.10	104	3.55 (3.45, 3.65)	0.17 (0.02, 0.33)	0.34^*^
Control	71	3.29 (3.18, 3.39)			58	3.37 (3.25, 3.50)			61	3.38 (3.25, 3.50)		
**SECONDARY OUTCOMES**												
IMRS clinician version												
Experimental	116	3.25 (3.18, 3.33)	0.05 (−0.07, 0.17)	0.12	99	3.45 (3.36, 3.55)	0.15 (0.01, 0.29)	0.33	105	3.53 (3.43, 3.62)	0.14 (−0.01, 0.30)	0.29
Control	71	3.20 (3.11, 3.3)			61	3.3 (3.2, 3.40)			57	3.38 (3.26, 3.51)		
**MSPSS**												
Experimental	116	5.04 (4.77, 5.31)	0.34 (−0.11, 0.79)	0.23	99	5.11 (4.83, 5.38)	0.41 (−0.05, 0.88)	0.28	104	5.24 (4.95, 5.53)	0.28 (−0.16, 0.72)	0.19
Control	70	4.70 (4.34, 5.06)			58	4.69 (4.31, 5.07)			61	4.96 (4.63, 5.29)		
**CSES**												
Experimental	116	137.16 (127.94, 146.38)	6.38 (−8.65, 21.41)	0.13	99	146.69 (138.87, 154.51)	15.1 (1.22, 28.98)	0.34	104	150.58 (142.07, 159.09)	14.43 (0.26, 28.60)	0.31
Control	71	130.78 (118.91, 142.65)			58	131.58 (120.11, 143.05)			61	136.14 (124.81, 147.48)		
**SES**												
Experimental	115	10.79 (10.44, 11.13)	0.0 (−0.57, 0.56)	0.00	99	10.73 (10.37, 11.09)	0.16 (−0.46, 0.79)	0.08	105	10.81 (10.41, 11.2)	−0.01 (−0.62, 0.61)	0.00
Control	71	10.79 (10.34, 11.23)			60	10.57 (10.06, 11.08)			56	10.82 (10.34, 11.29)		
**IS**												
Experimental	116	9.79 (9.33, 10.25)	0.39 (−0.52, 1.30)	0.14	99	9.83 (9.33, 10.33)	0.07 (−0.84, 0.99)	0.03	104	9.48 (8.88, 10.08)	−0.08 (−1.08, 0.91)	−0.03
Control	70	9.40 (8.62, 10.18)			57	9.75 (8.99, 10.52)			60	9.56 (8.76, 10.35)		
**ASI**												
Experimental	116	0.47 (0.25, 0.7)	0.02 (−0.31, 0.36)	0.02	99	0.27 (0.12, 0.42)	−0.09 (−0.34, 0.17)	−0.12	104	0.39 (0.14, 0.64)	−0.03 (−0.38, 0.31)	−0.03
Control	71	0.45 (0.2, 0.7)			58	0.36 (0.15, 0.57)			60	0.42 (0.18, 0.66)		
**BSI**												
Experimental	116	1.23 (1.07, 1.39)	−0.09 (−0.34, 0.16)	−0.11	99	1.15 (0.99, 1.3)	−0.18 (−0.44, 0.08)	−0.22	104	1.14 (0.98, 1.29)	−0.08 (−0.34, 0.17)	−0.10
Control	71	1.32 (1.13, 1.52)			58	1.33 (1.12, 1.54)			61	1.22 (1.02, 1.42)		
**SFS**												
Experimental	116	105.95 (104.33, 107.56)	1.08 (−1.44, 3.60)	0.12	99	106.56 (104.82, 108.3)	2.08 (−0.58, 4.73)	0.24	104	107.91 (106.2, 109.61)	1.9 (−0.85, 4.65)	0.21
Control	71	104.87 (102.93, 106.81)			58	104.48 (102.48, 106.49)			61	106.01 (103.85, 108.16)		
**MHRM**												
Experimental	116	70.15 (66.34, 73.96)	1.19 (−4.64, 7.03)	0.06	99	71.98 (68.13, 75.82)	4.24 (−1.88, 10.36)	0.21	104	74.05 (70.55, 77.56)	5.48 (−0.63, 11.58)	0.28
Control	71	68.96 (64.54, 73.38)			58	67.74 (62.98, 72.49)			61	68.58 (63.58, 73.58)		
**SERS-SF**												
Experimental	116	11.48 (7.18, 15.77)	4.57 (−2.46, 11.61)	0.19	99	14.74 (10.24, 19.24)	9.55 (2.85, 16.25)	0.43	104	17.64 (13.18, 22.11)	11.86 (4.95, 18.76)	0.52^*^
Control	71	6.91 (1.34, 12.48)			58	5.18 (0.22, 10.15)			61	5.79 (0.52, 11.05)		
**ISMI**												
Experimental	116	2.10 (2.00, 2.19)	−0.06 (−0.21, 0.10)	−0.11	99	2.02 (1.93, 2.11)	–−0.19 (−0.35, −0.04)	−0.41	104	2.01 (1.91, 2.11)	−0.10 (−0.26, 0.06)	−0.19
Control	71	2.16 (2.03, 2.28)			58	2.21 (2.08, 2.34)			61	2.11 (1.98, 2.23)		

* *A treatment effect was found on this variable as reported in Table 2*.

*IMRS, Illness Management and Recovery scale; MSPSS, Multidimensional Scale of Perceived Social Support; CSES, Coping Self-Efficacy Scale; SES, Service Engagement Scale, treatment adherence subscale; IS, Insight Scale; ASI, Addiction Severity Index, item 24; BSI, Brief Symptom Inventory; SFS, Social Functioning Scale; MHRM, Mental Health Recovery Measure; SERS-SF, Self-Esteem Rating Scale-Short Form; ISMI, Internal Stigma of Mental Illness*.

### Secondary Outcomes

Compared with the control group, the IMR group showed a statistically significant improvement in self-esteem (a component of personal recovery), which was measured using the SERS-SF (*p* = 0.01) (Table 2).

There were no effects on illness self-management measured via the clinician version of the IMR scale, on the illness management components of coping, social support, medication adherence, insight, and addiction, or on clinical, functional, and personal recovery measured via the MHRM (a composite measure) and the ISMI (a scale assessing self-stigma) (Table 2). The observed effect on self-esteem did not remain following Benjamini–Hochberg correction.

For all outcome measures, Table 3 shows the estimated marginal means (95% CI) from the LMM analyses. The results, including reported effect sizes (Cohen's *d*), concern the differences between the experimental and control condition (IMR vs. CAU) at different time points. Post-treatment, all measures showed improvement in favor of IMR with the exception of the IS and the ASI (Table 3). At follow-up, this improvement continued not only within the client version of the IMR scale, but also within the MHRM and SERS-SF personal recovery scales; those two scales demonstrated small and moderate effect-sizes, respectively.

Both the experimental and control group showed statistically significant improvement over time with respect to overall illness management, as measured using the clinician version of the IMR scale, as well as with respect to social support, clinical and functional recovery, and reduced self-stigma (Table 2).

After adjusting for hospitalization a year prior to T1, the treatment condition was not found to be a statistically significant predictor for hospitalization within the year following T2 [odds ratio (OR) = 1.22, 95% CI = 0.44, 3.42, *p* = 0.71). Regarding total days of hospitalization one year before and after treatment, there were no statistically significant between-group differences as measured by difference scores (IMR group, *M* = −7.72, SD = 51.19 vs. the control group, M = −4.46, SD = 44.23; U = 3,928.50, *p* = 0.50).

### Impact of Fidelity

Fidelity assessments via the IMR fidelity scale were conducted in 15 IMR groups, which enrolled 68 study participants who had attended ≥10 IMR sessions. This yielded a mean fidelity score of 3.94 (SD = 0.29). Eight groups (*n* = 39, 57%) had scores of ≥4 (range 4.00–4.54), which indicated high (54) or good (31) fidelity. Seven groups (*n* = 29, 43%) had scores <4 and >3 (range 3.46–3.92), which indicated moderate (54) or fair (31) fidelity. Lower fidelity scores were partly due to the fact that role-playing, a key element of cognitive-behavioral techniques and coping skills training, was practiced at a low rate within the evaluated interventions.

Assessment of the impact of fidelity on the results of the client version of the IMR scale as well as SERS-SF scores revealed no effects on overall IMR scale scores [F_(4, 271)_ = 1.42, *p* = 0.23]; however, we did observe a statistically significant overall interaction effect for group and time with respect to self-esteem [F_(4, 237)_ = 3.11, *p* = 0.02].

Examination of the fixed coefficients for the client version of the IMR scale revealed that there were no statistically significant differences in improvement between the high-fidelity and control groups at post-treatment (B = 0.08, *p* = 0.46) or at follow-up (B = 0.16, *p* = 0.08). Moreover, there were no statistically significant differences in improvement between the moderate fidelity and control groups at post-treatment (B = 0.06, *p* = 0.48); however, at follow-up, we did observe a statistically significant between-group difference in overall improvement (B = 0.20, *p* = 0.04) (Table 4).

**Table 4:** Fixed coefficients for linear mixed model (IMR client version) on time effects for high and moderate IMR fidelity.

**Variable**	**B**	**SE**	**T**	***p***	**95% CI**	
					**Lower**	**Upper**
Intercept	3.29	0.05	62.6	<0.001	3.18	3.39
**Group^a^**						
Experimental, high IMR fidelity	0.06	0.1	0.61	0.54	−0.13	0.25
Experimental, moderate IMR fidelity	0.05	0.11	0.41	0.68	−0.18	0.27
**Time^b^**						
Follow-up	0.09	0.06	1.52	0.13	−0.03	0.21
Post treatment	0.09	0.06	1.46	0.15	−0.03	0.20
**Group × time**						
Exp high fidelity at follow-up	0.16	0.09	1.78	0.08	−0.02	0.34
Exp high fidelity at post treatment	0.08	0.10	0.74	0.46	−0.13	0.28
Exp moderate fidelity at follow-up	0.20	0.10	2.05	0.04	0.01	0.38
Exp moderate fidelity at post treatment	0.06	0.09	0.71	0.48	−0.11	0.24

*IMR, Illness management and recovery scale*.

a *Reference category = control*.

b *Reference category = baseline*.

Regarding the fixed coefficients for the SERS-SF score, the high-fidelity subgroup showed a statistically significant improvement as compared with the control group at post-treatment (B = 7.22, *p* = 0.04) but not at follow-up (B = 4.96, *p* = 0.19). At post-treatment, the moderate fidelity subgroup did not improve at the level of statistical significance as compared with the control group (B = 2.65, *p* = 0.53); however, there was a statistically significant improvement at follow-up (B = 9.06, *p* = 0.01) (Table 5).

**Table 5:** Fixed coefficients for linear mixed model (SERS-SF) on time effects for high and moderate IMR fidelity.

**Variable**	**B**	**SE**	**T**	***p***	**95% CI**	
					**Lower**	**Upper**
Intercept	6.91	2.83	2.44	0.02	1.33	12.48
**Group^a^**						
Experimental, high IMR fidelity	3.21	4.81	0.67	0.51	−6.30	12.72
Experimental, moderate IMR fidelity	7.68	5.15	1.49	0.14	−2.48	17.84
**Time^b^**						
Follow-up	−1.15	2.42	−0.48	0.63	−5.90	3.60
Post treatment	−1.74	2.17	−0.80	0.42	−6.02	2.54
**Group × time**						
Exp high fidelity at follow-up	4.96	3.77	1.32	0.19	−2.45	12.36
Exp high fidelity at post treatment	7.22	3.40	2.12	0.04	0.50	13.93
Exp moderate fidelity at follow-up	9.06	3.61	2.51	0.01	1.96	16.16
Exp moderate fidelity at post treatment	2.65	4.20	0.63	0.53	−5.67	10.98

*SERS-SF, Self Esteem Rating Scale-Short Form*.

a *Reference category = control*.

b *Reference category = baseline*.

### IMR Completion

Analysis of overall illness management in completers using the client version of the IMR scale revealed a greater effect than that observed in the intention-to-treat analysis (*p* = 0.016), with small and moderate effect sizes at T2 and T3, respectively (for all outcomes for completers, see Tables 6, 7). Unlike analysis with the intention-to-treat principle, completer analysis within the clinician version of the IMR scale revealed a statistically significant result (*p* = 0.012), with large and moderate effect sizes at T2 and T3, respectively. For IMR completers, there were no effects on the five measured illness management components.

**Table 6:** Fixed effects of IMR on all outcomes from final linear mixed model, in the completers sample.

**Illness management and recovery scales**				**Illness management constituents**				**Clinical, functional and personal recovery**			
	**B**	**95% CI**	***p***		**B**	**95% CI**	***P***		**B**	**95% CI**	***p***
**IMRS client version^a^**				**Social support (MSPSS)**				**Clinical recovery (BSI)**			
Intercept	3.30	3.19 to 3.41	<0.001	Intercept	4.66	4.35 to 4.96	<0.001	Intercept	1.28	1.14 to 1.42	<0.001
Time	0.05	−0.01 to 0.09	0.09	Time	0.13	0.02 to 0.23	0.02	Time	−0.05	−0.10 to 0.003	0.07
Condition	0.09	−0.08 to 0.25	0.30	Condition	0.43	0.01 to 0.86	0.05	Condition^b^			
Condition × time	0.09	0.02 to 0.17	0.016^*^	Condition × time^b^				Condition × time^b^			
**IMRS clinician version**				**Coping self efficacy (CSES)**				**Functional Recovery (SFS)**			
Intercept	3.21	3.11 to 3.31	<0.001	Intercept	130.45	118.95 to 141.94	<0.001	Intercept	104.64	103.20 to 106.07	<0.001
Time	0.09	0.03 to 0.14	0.003	Time	1.95	−2.59 to 6.50	0.40	Time	0.93	0.38 to 1.47	0.001
Condition	0.13	0.02 to 0.27	0.08	Condition	2.29	−14.92 to 19.50	0.79	Condition^b^			
Condition × time	0.11	0.02 to 0.19	0.012^*^	Condition × time	5.72	−0.91 to 12.34	0.09	Condition x time^b^			
				**Medication adherence (SES)**				**Pers. recovery (MHRM)**			
				Intercept	10.92	10.67 to 11.17	<0.001	Intercept	68.80	64.27 to 73.32	<0.001
				Time^b^				Time	−0.65	−2.41 to 1.11	0.47
				Condition^b^				Condition	0.74	−6.03 to 7.51	0.83
				Condition × time^b^				Condition × time	2.87	−0.32 to 5.42	0.03^*^
				**Insight (IS)**				**Pers. recovery (SERS-SF)**			
				Intercept	9.82	9.39 to 10.25	<0.001	Intercept	6.75	1.44 to 12.06	0.01
				Time^b^				Time	−1.07	−3.37 to 1.22	0.36
				Condition^b^				Condition	5.78	−2.16 to 13.73	0.15
				Condition × time^b^				Condition × time	3.64	0.30 to 6.98	0.03^*^
				**Addiction (ASI)**				**Pers. recovery (ISMI)**			
				Intercept	0.38	0.23 to 0.52	<0.001	Intercept	2.17	2.06 to 2.28	<0.001
				Time^b^				Time	0.001	−0.05 to 0.05	0.97
				Condition^b^				Condition	−0.09	−0.27 to 0.08	0.28
				Condition × time^b^				Condition × time	−0.05	−0.12 to 0.01	0.12

a *Primary outcome measure*.

b *These cells remain empty because, with stepwise modeling, “the best fitting model” was found at an earlier step*.

* *A statistically significant interaction effect, p < 0.05. IMRS, Illness Management and Recovery scale; MSPSS, Multidimensional Scale of Perceived Social Support; CSES, Coping Self-Efficacy Scale; SES, Service Engagement Scale, subscale treatment adherence; IS, Insight Scale; ASI, Addiction Severity Index, item 24; BSI, Brief Symptom Inventory; SFS, Social Functioning Scale. MHRM, Mental Health Recovery Measure; SERS-SF, Self-Esteem Rating Scale-Short Form; ISMI, Internal Stigma of Mental Illness*.

**Table 7:** Estimated outcomes as a function of IMR, estimated from linear mixed models in the completers sample.

	**Baseline**				**Post treatment (12 months)**				**Follow-up (18 months)**			
			**Mean difference**				**Mean difference**				**Mean difference**	
	***N***	**Mean (95% CI)**	**(95% CI)**	***d***	***N***	**Mean (95% CI)**	**(95% CI)**	***d***	***N***	**Mean (95% CI)**	**(95% CI)**	***d***
**PRIMARY OUTCOME**												
**IMRS client version**												
Experimental	57	3.36 (3.23, 3.50)	0.08 (−0.09, 0.25)	0.16	53	3.56 (3.43, 3.69)	0.19 (0.01, 0.37)	0.38	54	3.65 (3.53, 3.77)	0.27 (0.10, 0.44)	0.59^*^
Control	71	3.29 (3.18, 3.39)			58	3.37 (3.24, 3.50)			58	3.38 (3.26, 3.50)		
**SECONDARY OUTCOMES**												
**IMRS clinician version**												
Experimental	57	3.29 (3.19, 3.39)	0.08 (−0.05, 022)	0.21	53	3.63 (3.52, 3.74)	0.33 (0.18, 0.48)	0.79	54	3.67 (3.55, 3.79)	0.29 (0.12, 0.47)	0.62^*^
Control	71	3.20 (3.11, 3.30)			61	3.30 (3.20, 3.41)			54	3.37 (3.24, 3.50)		
**MSPSS**												
Experimental	57	5.02 (4.65, 5.39)	0.32 (−0.19, 0.84)	0.22	53	5.28 (4.97, 5.59)	0.56 (0.08, 1.05)	0.43	54	5.33 (4.99, 5.67)	0.43 (−0.05, 0.91)	0.33
Control	70	4.70 (4.34, 5.06)			58	4.72 (4.34, 5.09)			58	4.90 (4.57, 5.24)		
**CSES**												
Experimental	57	131.04 (117.96, 144.11)	0.26 (−17.41, 17.92)	0.01	53	144.12 (133.07, 155.17)	12.72 (−3.23, 28.68)	0.28	54	146.19 (134.75, 157.64)	11.51 (−4.58, 27.60)	0.26
Control	71	130.78 (118.91, 142.65)			58	131.40 (119.89, 142.90)			58	134.68 (123.37, 145.99)		
**SES**												
Experimental	56	11.09 (10.62, 11.56)	0.30 (−0.34, 0.95)	0.16	53	11.18 (10.81, 11.55)	0.60 (−0.03, 1.24)	0.34	54	11.26 (10.81, 11.71)	0.53 (−0.13, 1.20)	0.31
Control	71	10.79 (10.34, 11.24)			60	10.57 (10.06, 11.09)			54	10.72 (10.24, 11.21)		
**IS**												
Experimental	57	10.06 (9.39, 10.73)	0.64 (−0.39, 1.67)	0.21	53	10.18 (9.61, 10.76)	0.39 (−0.56, 1.35)	0.15	54	10.07 (9.35, 10.78)	0.48 (−0.61, 1.56)	0.16
Control	70	9.41 (8.63, 10.20)			57	9.79 (9.02, 10.56)			57	9.59 (8.77, 10.41)		
**ASI**												
Experimental	57	0.40 (0.14, 0.67)	−0.05 (−0.41, 0.32)	−0.04	53	0.34 (0.10, 0.59)	0.00 (−0.32, 032)	0.00	54	0.29 (−0.03, 0.60)	−0.11 (−0.50, 0.28)	−0.11
Control	71	0.45 (0.20, 0.70)			58	0.35 (0.15, 0.54)			57	0.40 (0.16, 0.63)		
**BSI**												
Experimental	57	1.23 (1.03, 1.44)	−0.09 (−0.37, 0.20)	−0.11	53	1.10 (0.88, 1.31)	−0.23 (−0.53, 0.07)	−0.27	54	1.12 (0.91, 1.33)	−0.12 (−0.42, 0.17)	−0.15
Control	71	1.32 (1.12, 1.52)			58	1.33 (1.12, 1.54)			58	1.24 (1.04, 1.45)		
**SFS**												
Experimental	57	104.48 (102.19, 106.77)	−0.39 (−3.39, 2.62)	−0.05	53	106.42 (104.25, 108.58)	1.92 (−1.03, 4.87)	0.23	54	107.31 (106.13, 109.49)	1.45 (−1.64, 4.53)	0.17
Control	71	104.87 (102.92, 106.81)			58	104.50 (102.49, 106.50)			58	105.86 (103.68, 108.05)		
**MHRM**												
Experimental	57	69.04 (63.43, 74.65)	0.08 (−7.07, 7.23)	0.00	53	72.86 (67.33, 78.39)	5.15 (−2.15, 12.45)	0.25	54	73.48 (69.02, 77.94)	5.79 (−0.86, 12.45)	0.32^*^
Control	71	68.96 (64.53, 73.40)			58	67.71 (62.94, 74.49)			58	67.68 (62.75, 72.62)		
**SERS-SF**												
Experimental	57	11.76 (5.80, 17.72)	4.85 (−3.31, 13.01)	0.16	53	16.81 (10.76, 22.85)	11.54 (3.70, 19.38)	0.53	54	16.88 (10.60, 23.16)	12.13 (4.01, 20.26)	0.54^*^
Control	71	6.91 (1.33, 12.49)			58	5.72 (0.27, 10.26)			58	4.75 (−0.42, 9.91)		
**ISMI**												
Experimental	57	2.10 (1.97, 2.22)	−0.06 (−0.24, 0.12)	−0.12	53	1.98 (1.86, 2.10)	−0.23 (−0.40, −0.06)	−0.48	54	1.99 (1.86, 2.12)	−0.17 (−0.35, 0.01)	−0.34
Control	71	2.16 (2.03, 2.28)			58	2.21 (2.08, 2.33)			58	2.16 (2.03, 2.28)		

* *A treatment effect was found on this variable as reported in Table 6*.

*IMRS, Illness Management and Recovery scale; MSPSS, Multidimensional Scale of Perceived Social Support; CSES, Coping Self-Efficacy Scale; SES, Service Engagement Scale, subscale treatment adherence; IS, Insight Scale; ASI, Addiction Severity Index, item 24; BSI, Brief Symptom Inventory; SFS, Social Functioning Scale; MHRM, Mental Health Recovery Measure; SERS-SF, Self-Esteem Rating Scale-Short Form; ISMI, Internal Stigma of Mental Illness*.

**Figure 3:**
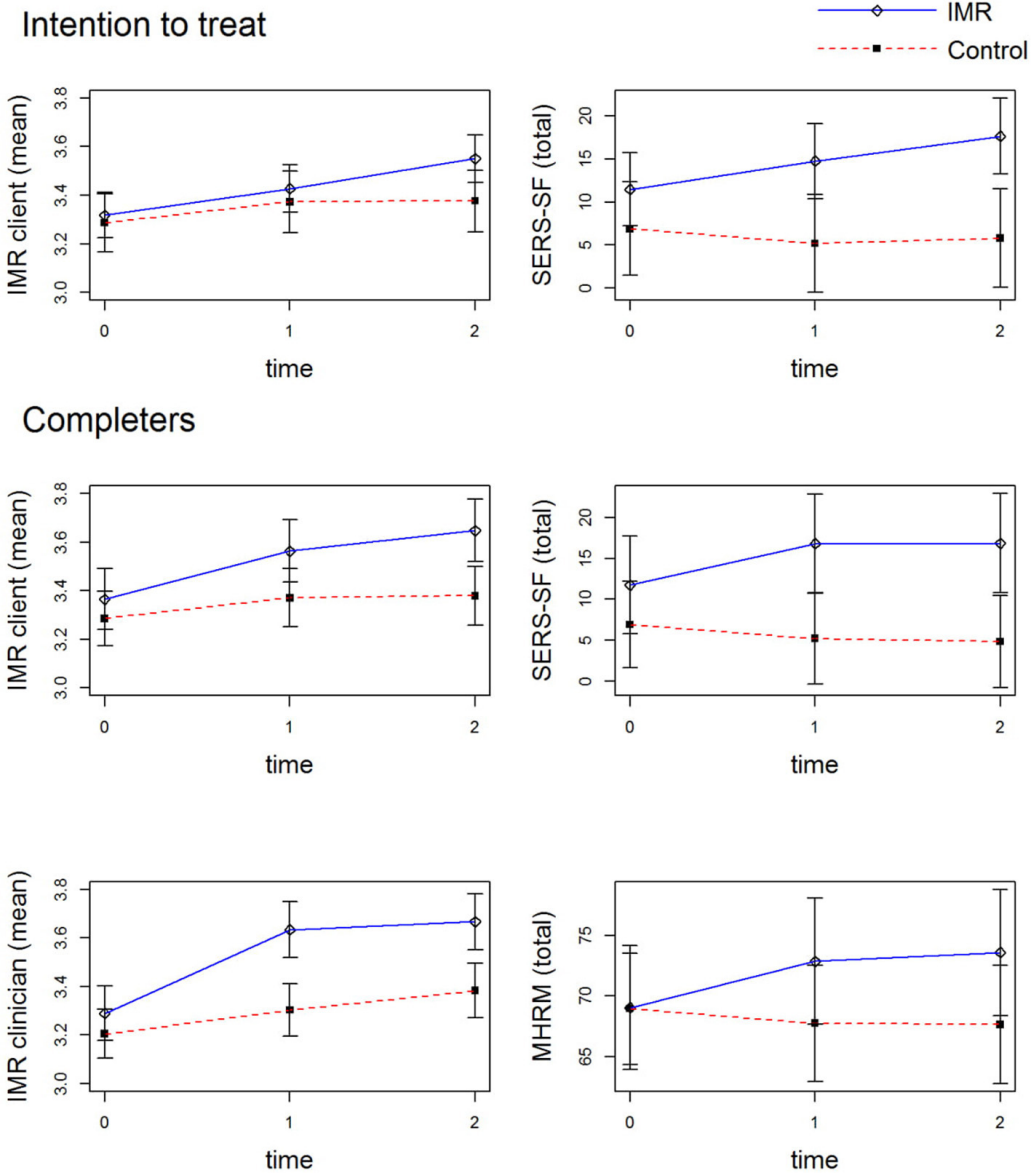
Significant Outcomes at 18 Months. 0 = baseline measurement, 1 = 12-months measurement, 2 = 18-months measurement. IMR client, client version of the Illness Management and Recovery scale; IMR clinician, clinician version of the Illness Management and Recovery scale; SERS-SF, Self Esteem Rating Scale-Short Form; MHRM, Mental Health Recovery Measure.

With respect to self-esteem, similar to the findings of the intention-to-treat analysis IMR completers showed statistically significant improvement as compared with the control group (*p* = 0.03) with moderate effect sizes at T2 and T3. Unlike in the intention-to-treat analysis, IMR completers showed a statistically significant improvement in overall personal recovery measured via the MHRM (*p* = 0.03), with small effect sizes at T2 and T3 (for all statistically significant outcomes at 18 months, see Figure 3). For IMR completers, there was no effect on self-stigma. Further, similar to the intention-to-treat analysis, IMR completion did not show effects on clinical and functional recovery.

The possibility of selective IMR non-completion was explored using 12 baseline characteristics. It was found that selective non-completion of IMR was unlikely, as suggested by multivariate logistic regression analysis [omnibus test: $\chi_{\left(13\right)}^{2}$ = 11.64, *p* = 0.56; Supplementary Table 1].

## Discussion

### Discussion of the Results

Our positive findings regarding the client version of the IMR scale, an overall measure of illness self-management and our primary outcome measure, confirms the effectiveness of IMR as compared with CAU. Additionally, our findings in the sensitivity analysis for IMR completers regarding the effects on both the client and clinician versions of the IMR scale support this positive finding.

Although our results with respect to these IMR scales indicate an overall improvement in illness management, the questionnaire scores on specific components of illness management revealed no statistically significant effects. These questionnaire scales assessed social support, coping, medication adherence, insight, and addiction. Although the IMR scales capture the overall changes in these components, IMR training may have insufficient specificity for generating meaningful effects on individual measures within these separate domains.

Additionally, as compared with the control group, the intervention group showed a greater improvement in self-esteem. This positive outcome is supported by the findings for the sensitivity analysis among IMR completers. Compared with the control group, IMR completers had superior outcomes with respect to self-esteem and the MHRM score, a composite measure of personal recovery. These combined results indicate the potential efficacy of IMR with respect to personal recovery.

In contrast, the IMR intervention did not show a statistically significant effect on psychiatric symptoms. This is consistent with the findings of a recent meta-analysis that indicated a small to moderate association between clinical and personal recovery in patients with schizophrenia-spectrum disorders (15). Furthermore, clinical recovery does not appear to be necessary for personal recovery (11, 15).

Previous studies have confirmed the relevance of improvement in personal recovery by demonstrating the importance of the therapeutic benefits of managing mental illness, which facilitates personal well-being and self-perceived growth. Additionally, studies have highlighted the value of living a satisfying life despite the presence of enduring symptoms (13, 19, 65, 66). Therefore, changes should be measured based on both symptomatology and consumer-defined recovery (5, 20, 65, 67). Apart from symptoms (13), loss of self-esteem is considered to greatly affect the life and self-image of patients with schizophrenia (53, 57, 68). The concept of self-esteem corresponds with the concept of identity (i.e., a positive sense of self), which is one of the five processes within the CHIME conceptual framework of personal recovery (20).

The impact of fidelity was analyzed within the two scales, revealing statistically significant outcomes in the intention-to-treat analysis. There was no effect of fidelity with respect to the client version of the IMR scale. However, the fidelity of IMR training affected SERS-SF scores. This suggests that fidelity is relevant to the effectiveness of IMR with respect to self-esteem. However, there is a need to further research the predictive validity of the IMR fidelity scale within high-powered gold-standard investigations.

Although our positive results regarding the client version of the IMR scale are consistent with the results of three RCTs on IMR (22, 23, 28), they differ from those of three other RCTs (21, 24–27). Moreover, our negative results regarding the clinician version of the IMR scale are consistent with those of a previous RCT (25, 27); however, they differ from the results of four previous RCTs reporting positive outcomes (21–23, 28, 29). Our positive findings regarding personal recovery differ from those of three previous RCTs (23–25, 27). This inconsistency in results may be attributed at least in part to differing completion rates between the investigations. We thereby suggest that the differing attendance rates within IMR may have affected the findings of this and other RCTs with respect to IMR outcomes.

A previous RCT that reported no statistically significant effects was conducted among a cohort with an IMR attendance of only 28% (24). Two of three RCTs with IMR completion rates of ~50% (including our study) reported positive effects (22), while the third RCT reported null results (25–27). Two RCTs with completion rates of 100% reported positive effects on psychiatric symptoms as well as on both IMR scales (23, 28). Additionally, Levitt et al. found that, with respect to the intention-to-treat analysis, the effect size for completers increased from 0.36 to 0.75 and from 0.39 to 0.59 on the client and clinician versions of the IMR scale, respectively (22). This is consistent with our findings, wherein effect sizes on both IMR scales substantially increased in the completer subgroup.

A minimum threshold of exposure to IMR could be required for treatment effects to occur (24). Therefore, in addition to the percentage of participants exposed to >50% of the scheduled sessions, the total number of sessions attended is also relevant in evaluating treatment efficacy. In the current study, the mean attendance for the IMR group was 23.57 sessions (SD = 21.09), with completers attending an average of 42.40 sessions (SD = 12.53). A Danish RCT (25–27) that did not observe any effects had an average attendance of 16.4 sessions; moreover, completers (defined as participants exposed to >10 sessions) had only attended an average of 26.1 sessions. Therefore, the results of the two negative trials on IMR (24–27) could be partly attributed to their respective low completion rates.

The importance of a higher completion rate with respect to the efficacy of IMR suggests the relevance of increasing the motivation to continue IMR among patients. In addition to reminders and phone calls, IMR trainers should employ other strategies for facilitating attendance (24). For example, to promote the efficient use of available places at our study sites, the current study had rolling admission into IMR groups; however, peer support—and thus participation—may be promoted if groups have a closed enrollment format (24). At one site in our study, IMR groups had lunch together at each meeting in order to promote group cohesion and thus attendance. In addition, to promote completion, the two respective RCTs on IMR that had the highest percentages of completers selected participants based on earlier treatment adherence (23, 24) and applied the total IMR curriculum using home-visits (28). However, choosing one or more of these options will not be preferred or feasible at all practices and centers.

Although our study demonstrated positive results, the observed effects were small. We observed a statistically significant effect on illness self-management within the client version of the IMR scale (*p* = 0.048). Although we found effects on personal recovery, there were limited effects on other secondary outcome measures in the current study. These limitations could be attributed to several potential reasons. First, IMR training may not be sufficiently specific for generating statistically significant effects on the separate components of illness self-management. This limitation may be associated with the multiplicity of IMR objectives, as shown in the previously published conceptual framework for IMR (3). Second, participants in both experimental conditions showed improvement in five domains over time as follows: clinician-rated overall illness management, social support, clinical and functional recovery, and self-stigma. These findings may be attributed to the positive effects of CAU, given that many clinicians in the Netherlands have been trained in psychiatric rehabilitation methodologies, including the Boston University Approach to Psychiatric Rehabilitation (69). Additionally, standard outpatient treatment and care have evolved into the FACT model. FACT (Function ACT) is a rehabilitation-oriented clinical case management model; a flexible version of ACT (70). Third, in our study, most of the IMR practitioners (community mental health nurses and social workers) had no specific training in the empirically supported strategies underlying IMR, including cognitive-behavioral approaches and skills training. Thus, while the facilitators may have had training in rehabilitation skills and attitudes, other specific clinical skills required for the successful implementation of IMR were often insufficient. Moreover, with respect to IMR group training, it may be challenging to provide these EBPs based on the required protocols. This challenge was partly reflected in the observed fidelity scores.

Our study observed improved outcomes in only one of the eleven secondary outcomes in the intervention group. Sensitivity analyses for completers revealed improved outcomes in the intervention group for four of the eleven secondary outcomes. These effects were not observed following Benjamini-Hochberg correction for multiple testing. However, multiple adjustment methodologies have been criticized within the statistical, epidemiological, and medical literature (63, 71). For example, some researchers have indicated that multiple correction methodologies mechanize, and therefore trivialize, interpretative problems. In addition, multiplicity adjustments are not considered appropriate for more exploratory secondary analyses (63, 71). Therefore, we suggest that the present interpretation of our findings may be relevant.

### Strengths and Limitations

Our study has five main strengths. First, our sample size was relatively large and we used a complete set of outcomes. This allowed for rigorous measurement of the IMR effects. Second, we thoroughly investigated the impact of completion rates. Third, to our knowledge, this is the first RCT on IMR to assess the impact of fidelity. Fourth, this is the first RCT on IMR to find effects on personal recovery. Finally, given the natural setting of the current investigation, our results can be assumed to have good generalizability.

In addition to these substantial strengths, this study had several limitations. First, there was suboptimal IMR implementation, with approximately half of the IMR-participants completing the program; furthermore, IMR fidelity was only fair to moderate for almost half of the IMR participants. For example, skills training using role play was applied at a low rate within the evaluated interventions. This suboptimal IMR implementation may have led to an underestimation of the outcomes. Second, since we aimed to comprehensively explore the effects of IMR, we examined numerous secondary outcome measures. Therefore, with reference to alpha inflation, the statistically significant result regarding self-esteem can be disputed. Third, when completing the clinician version of the IMR scale and the Service Engagement Scale, clinicians were not blinded to their patients' experimental conditions. Since we used self-score questionnaires, the patients were not blinded to the treatment condition. However, all interviewers were blinded to the condition. Fourth, in measuring the impact of fidelity, we could only utilize the fidelity scores of the 68 participants in the 15 IMR groups. This limits the generalizability of our results. Fifth, most of the enrolled participants had relatively few problems in the three domains of addiction, medication adherence, and insight at baseline; therefore, there was little room for improvement within these domains.

### Conclusions

In the current study, we observed positive results with respect to the client version of the IMR scale. Our results support the effectiveness of IMR in overall illness self-management. This finding was confirmed within our secondary analysis among IMR completers. However, we observed negative results for five specific components of illness management. Therefore, our findings suggest that IMR is a non-specific intervention for illness self-management.

This study provides indications regarding the efficacy of IMR on components of personal recovery. However, we found no effects on clinical and functional recovery. There is a need for further research to confirm these findings.

We suggest that challenges in implementing the multiple specific clinical skills required for IMR may impede the achievement of specific outcomes. Therefore, more training as well as more specific and comprehensive training in different IMR elements is recommended based on the results of this research. However, within IMR, protocolized application of the EBPs underlying IMR may present a logistical challenge.

The quality of IMR implementation appeared to be relevant given the association of IMR fidelity with effects on self-esteem; moreover, greater exposure to IMR appeared to enhance these effects. Therefore, based on the findings of the current research as well as the overall literature to date, we propose more comprehensive initial and continuing education for IMR trainers in order to improve high-fidelity IMR implementation, as well as an emphasis on new and innovative strategies focusing on promoting IMR completion.

## Data Availability Statement

The datasets presented in this article are not readily available because Parnassia Groep Mental Health Care needs to consent to data access. Requests to access the datasets should be directed to Bert-Jan Roosenschoon, b.roosenschoon@parnassiagroep.nl.

## Ethics Statement

The studies involving human participants were reviewed and approved by the Research Ethics Committee (METC) of the Erasmus MC University Medical Center Rotterdam. The patients/participants provided their written informed consent to participate in this study.

## Author Contributions

B-JR, CM, JW, and MD conceived and designed the study. B-JR managed recruitment, data collection, data administration, and drafted the article, which was critically revised by all authors. EE coordinated data collection and supervised the quality of IMR at Yulius Mental Health. B-JR and MD did statistical analysis. B-JR, CM, JW, MD, and AK had full access to interim reports on statistics, analyses, and tables. All authors contributed to the article and approved the submitted version.

## Funding

This work was supported mainly by the Parnassia Groep Psychiatric Institute (The Hague/ Rotterdam, the Netherlands); it was also supported by Janssen-Cilag B. V. with additional funding of an unconditional educational grant (1-4H1ZDZ67890). The funding organizations had no role in the study design, data collection, data analysis, data interpretation, writing of the report, or the decision to publish the study.

## Conflict of Interest

The authors declare that the research was conducted in the absence of any commercial or financial relationships that could be construed as a potential conflict of interest.

## Publisher's Note

All claims expressed in this article are solely those of the authors and do not necessarily represent those of their affiliated organizations, or those of the publisher, the editors and the reviewers. Any product that may be evaluated in this article, or claim that may be made by its manufacturer, is not guaranteed or endorsed by the publisher.

## Supplementary Material

Supplementary Table 1 Coefficients, odds ratios and *CI*'s, predicting non-completion from baseline characteristics.

Supplementary Data Sheet 1 Positive decision Erasmus MC.

Supplementary Data Sheet 2 Original study Protocol for the METC.
