# Supplementary material for: Effects of Illness Management and Recovery: A Multicenter Randomized Controlled Trial
Source: Front Psychiatry. 2021 Dec 14;12:723435. doi: 10.3389/fpsyt.2021.723435 (PMC8712643; doi:10.3389/fpsyt.2021.723435)
Supplement: Supplementary Table 1 — Coefficients, odds ratios and CI's, predicting non-completion from baseline characteristics. [file Table_1.DOCX]

| **Supplementary Table 1** Coefficients, odds ratios and *CI’*s, predicting non-completion from baseline  characteristics | | | | | | |
| --- | --- | --- | --- | --- | --- | --- |
|  | *B* | *SE(B)* | *p* | *OR* | 95% *CI* for *OR* | |
|  |  |  |  |  | Lower | Upper |
| Intercept | 4,385 | 2,562 | 0,087 | - | - | - |
| Age | -0,027 | 0,021 | 0,195 | 0,973 | 0,934 | 1,014 |
| Sex | -0,033 | 0,409 | 0,936 | 0,968 | 0,434 | 2,157 |
| Education level^a^ |  |  | 0,975 |  |  |  |
| middle | -0,109 | 0,487 | 0,823 | 0,897 | 0,345 | 2,328 |
| high | -0,055 | 0,546 | 0,919 | 0,946 | 0,324 | 2,761 |
| Diagnosis  Psychotic disorders | -0,166 | 0,593 | 0,780 | 0,847 | 0,265 | 2,707 |
| Mood disorders | -0,735 | 0,603 | 0,223 | 0,480 | 0,147 | 1,563 |
| Personality disorders | -0,534 | 0,501 | 0,287 | 0,586 | 0,219 | 1,567 |
| Start of problems > 10 years previous to IMR | -0,051 | 0,454 | 0,911 | 0,950 | 0,390 | 2,315 |
| IMRS client version | -1,149 | 0,600 | 0,055 | 0,317 | 0,098 | 1,027 |
| IMRS clinician version | -0,097 | 0,554 | 0,862 | 0,908 | 0,306 | 2,690 |
| CSES | 0,013 | 0,007 | 0,076 | 1,013 | 0,999 | 1,028 |
| BSI | -0,014 | 0,301 | 0,964 | 0,987 | 0,547 | 1,780 |
| MHRM | 0,004 | 0,018 | 0,840 | 1,004 | 0,969 | 1,039 |
| Note: ^a^ reference category = low; IMRS= Illness Management and Recovery Scale; CSES= Coping Self Efficacy Scale; BSI= Brief Symptom Inventory; MHRM= Mental Health Recovery Measure. | | | | | | |
| . | | | | | | |
