# Supplementary material for: Effects of Illness Management and Recovery: A Multicenter Randomized Controlled Trial
Source: Front Psychiatry. 2021 Dec 14;12:723435. doi: 10.3389/fpsyt.2021.723435 (PMC8712643; doi:10.3389/fpsyt.2021.723435)

ParnassiaBavo Academie

bavo europoort
specialist in psychiatrie

# Protocol

## Trial Illness Management and Recovery (IMR) in Bavo Europoort

### Effects of IMR on patients with severe mental illness

NL 38605, Versie 2

July 5th 2012

Drs. B.J. Roosenschoon
Prof. Dr. C.L. Mulder
Prof. Dr. J. van Weeghel

**PROTOCOL TITLE :** Trial Illness Management and Recovery (IMR) in Bavo Europoort

| | |
|---|---|
| Protocol ID | NL 38605 |
| Short title | Trial IMR Bavo Europoort |
| Version | 2 |
| Date | February 24 th 2012 |
| Coordinating investigator/project leader | Prof.dr.C.L.Mulder, Erasmus MC, O3 Mental Health Care Research Center , Department of Psychiatry Postbus 2040, 3000 CA Rotterdam c.l.mulder@erasmusmc.nl |
| Principal investigator(s) (in Dutch: hoofdonderzoeker/uitvoerder) *Multicenter research: per site* | Drs. B.J Roosenschoon, Parnassia Bavo Academie afd. Wetenschappelijk Onderzoek Monsterseweg 83, 2553 RJ Den Haag b.roosenschoon@parnassiabavogroep.nl |
| Sponsor (in Dutch: verrichter/opdrachtgever) | Bavo Europoort BV, Wim van Beek, directeur Prins Constantijnweg 48-54, 3066TA Rotterdam w.vanbeek@parnassiabavogroep.nl |
| Independent physician(s) | Stien Wolters psychiater, Bavo Europoort, Oudedijk 76 3062 AG Rotterdam s.wolters@parnassiabavogroep.nl |

**PROTOCOL SIGNATURE SHEET**

| Name | Signature | Date |
|---|---|---|
| Sponsor or legal representative: For non-commercial research, Head of Department: Prof.dr. W.J.J. Hoogendijk, Hoogleraar Neurobiologische Psychiatrie EMC | | |
| Principal Investigator: Drs. B.J. Roosenschoon - Senior onderzoeker Parnassia Bavo Academie - Onderzoeker/ Promovendus O3/ afd. Psychiatrie EMC | | |

## Index


| | page |
|---|---|
| 1. Background | 5 |
| 1.1. Problem definition | 5 |
| 1.2 Relevance | 7 |
| 1.2.1 Strengthening demand-orientation | 7 |
| 1.2.2 Practical significance | 9 |
| 1.2.3 Scientific significance | 9 |
| 2. Objective | 9 |
| 3. Research questions | 9 |
| 4. Design | 9 |
| 5. Study population | 10 |
| 5.1 Patients | 10 |
| 5.2 Exclusion criteria | 11 |
| 5.3 Sample Size | 11 |
| 6. Intervention | 11 |
| 7. Intervention- and control group | 11 |
| 8. Methods | 12 |
| 8.1 Outcome measures | 12 |
| 8.1.1 Primary outcome measures | 12 |
| 8.1.2 Secondary outcome measures | 13 |
| 8.1.3 Exploratory analysis | 15 |
| 8.2 Procedures | 15 |
| 8.2.1 Selection of patients | 15 |
| 8.2.2 Training of clinicians | 16 |
| 8.2.3 Recruitment of patients | 16 |
| 8.2.4 Research data | 16 |
| 8.2.5 Organization of data collection | 17 |
| 8.3 Participating sites | 17 |

9. Statistics 17

10. Feasibility of the study 18

11. Time frame 18

12. Ethical considerations 19

12.1 Recruitment and informed consent 19
12.2 Randomization and refusal 19

13. Funding 19

*Planned papers* 19

*Literature* 20

Annex 1 Flow chart of randomisation of participants 24

Annex 2 Budget Trial IMR at Bavo Europoort 25

Annex 3 Overview instruments + research contacts per patient 26

Annex 4 Flowchart researchprotocol Trial Illness Management and Recovery (IMR) in Bavo Europoort 27

# 1. Background

## 1.1. Problem definition

*Introduction*

In the past care for people with serious and persistent psychiatric illnesses (SMI) had many shortcomings with regard to the quality and the availability of services. In recent years some promising new services have emerged, mostly in the USA, but their effectiveness in Dutch Mental Health Care has not yet been proven.
Examples include psychosocial interventions such as psycho-education, cognitive behavioral therapy, skills training, peer support, rehabilitation care and interventions supporting recovery. These interventions can help patients to get a better grip on their problems (Illness management) and can promote recovery, but are often not available. Therefore several of these psychosocial interventions are at a basic level included in the program of Illness Management and Recovery (IMR), in Dutch: 'Ziektemanagement en Herstel' or 'Hersteltraining'.
There is some evidence that IMR helps patients to gain control over their illness and to achieve their personal goals (Hasson-Ohayon e.a., 2007; Levitt et al. 2009, Färdig R. et al. 2011).
If desired, subsequent to the IMR program patients receive additional psychosocial treatments. The different parts of the IMR program are not new in Dutch Mental Health Care, but what is new is to offer these services together as an integrated package.
IMR is in line with the goals of patient organizations, which have made recovery a central concept. IMR aims to assist in their goals – to empower patients to take control of their illness and their lives, and work personally for their recovery.
According to Mueser et al. (2002a) IMR is linked to concepts such as recovery, hope, empowerment, personal freedom, cooperation, respect, recognition of the patient as an expert within his own experiences with mental health and 'illness management'.

*Phase of implementation of IMR*

IMR is currently implemented in several countries. In the Netherlands there is much interest in this type of care. In the Netherlands BavoEuropoort has the most experience with IMR. In some other institutions implementation of IMR is still at the beginning. Mental health care institution 'Rivierduinen' has some experience with IMR (Bovenberg et al 2006, Bovenberg & Staats 2008). In our organisation (BavoEuropoort) from 2009 on, IMR has been implemented on a wider scale (Ebbers 2008), and now 12 groups have completed an IMR course. These groups completed the IMR course after an average period of 12.6 months.

The implementation of IMR at BavoEuropoort has been evaluated with a pilot study, which aims to provide guidelines for an optimal implementation of IMR. This pilot study was also intended as a preparation for the RCT. The report of the pilot will be completed during the course of 2012. Some results are:

• Completers seem to benefit from IMR. • More women complete IMR
• Completers at baseline score significantly better than non-completers on the IMR-scale client version, IMR-scale clinicians version and on the GAF (psychosocial functioning)
• The fidelity of implementation varies by group and appears to be related to the quality of the trainers.
• The supervision of the trainers needs a substantial boost.
• There is a high rate of drop-out from treatment, especially in the stages prior to the start of the IMR Group.
• The participants who at the start score better on the IMR-scales have a relatively lower drop-out.
• Recruitment of participants in IMR is not easy.
• Given the successful progress of the pilot study it seems that the RCT-study is quite feasible. Data collection in the pilot study went relatively smoothly.

*Status of IMR as an Evidence-Based Practice (EBP)*

The IMR program was developed in the United States as part of the National Implementing Evidence-Based Practices Project, a major project to implement six evidence-based care programs in the U.S (Drake et al., 2001; Mueser et al., 2003). Other EBP-programs in this project are Assertive Community Treatment (ACT), Integrated Dual Disorder Treatment (IDDT), Individual Placement and Support (IPS), Family Psycho Education (FPE) and Medication Management (MM). With evidence-based we mean that research has given conclusive support that the treatment type is effective.

IMR was based on an empirical review of the research literature on teaching illness self-management strategies to persons with severe mental illness (Mueser et al., 2002). This review identified five empirically supported interventions which were subsequently incorporated into the IMR program: psycho-education, cognitive-behavioral approaches to medication, adherence, teaching relapse prevention, social skills training to improve social support, and coping skills training for persistent symptoms. IMR is named an EBP by the American researchers who have constructed IMR because the ingredients of the program are evidence-based. However, critics have expressed concerns that too many potentially good interventions are given in a short time, with the implication that none of them can be properly implemented (Van der Gaag 2008). For example, to implement cognitive-behavioral therapy (CBT) appropriately, a systematic protocol applied by well-trained staff would be necessary. Moreover critics have pointed to a lack of evidence that social and coping skills programmes are effective. (see the Dutch Multidisciplinary Guidelines for Schizophrenia, 2005). Since this date, however, three RCT's on the overall package of IMR have been executed by Hasson-Ohayon et al (2007), Levitt et al (2009) and Färdig et al. (2011), with positive results for IMR. Despite these positive studies, IMR is not yet broadly accepted as an EBP in Dutch mental health care. In particular, it is mentioned neither in the Dutch multidisciplinary guideline for schizophrenia of 2005, nor in the concept guideline of 2010. More research is needed, especially to investigate whether IMR is effective in the Dutch context.

*Theoretical base of IMR*

For the theoretical base of IMR is cited from Mueser et al. (2006):

"The IMR program integrates specific empirically supported strategies for teaching illness self-management into a cohesive treatment package based on 2 theoretical models: the trans-theoretical model and the stress-vulnerability model.

The trans-theoretical model proposes that motivation to change develops over a series of stages (pre-contemplation, contemplation, preparation, action, maintenance) and that facilitating change requires stage-specific interventions (Prochaska & DiClemente 1984; Prochaska 1984).

At the earliest stages, people are not committed to change and intervention focuses on instilling motivation. In the IMR program, motivational interviewing (Corrigan et al. 2001; Miller & Rollnick 2002) is used at the beginning and throughout the program to help clients develop their own vision of recovery, to identify and pursue their personal goals based on that vision, and to explore how improved illness management can help them achieve these goals.

The stress-vulnerability model (Liberman et al. 1986; Zubin & Spring 1977) posits that the course and outcome of schizophrenia is determined by the dynamic interplay of biological vulnerability, stress, and coping. IMR is aimed at interrupting the cycle of stress and vulnerability that leads to relapse and poor functioning (see figure 1). In IMR, the proximal goal is to teach clients the fundamentals of illness self-management based on the stress-vulnerability model (ie, adherence to medications, reduced substance use, increased social support, increased coping, involvement in meaningful activities) in order to improve illness outcomes such as symptoms, relapses, and hospitalizations. Then, through the combination of pursuing personal goals and improved illness self-management, the distal goal of IMR is to help clients make progress toward recovery, including objective (e.g. community functioning, social relationships, work) and subjective (e.g. sense of purpose, hope, confidence) dimensions."

**Conceptual Framework for the Illness Management and Recovery program**

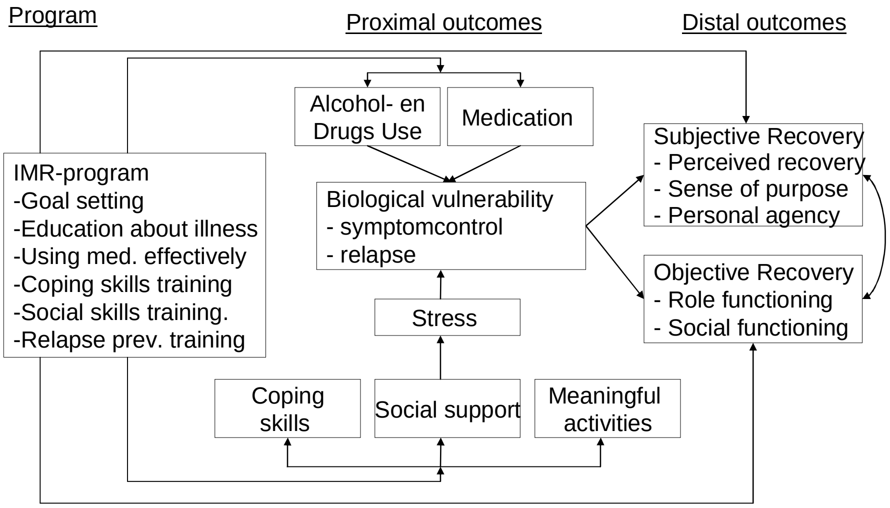


Figure 1 source: (Mueser et al. 2006)

## 1.2 Relevance

### 1.2.1 Strengthening demand-orientation

*What are the main elements of IMR?*

Illness Management and Recovery (IMR) is a program of care in the form of a training course for patients with severe and persistent psychiatric problems, based on a combination of different types of interventions focused on recovery. The idea underlying the training course is that the patient learns to gain control of his illness (illness management) and to make appropriate choices based on accurate information and skills training.

IMR can be described as a structured training program for people with severe and persistent mental illness which helps to:

- Set meaningful personal goals
- To gather information and acquire skills to achieve a greater degree of control over the psychiatric illness.
- To work on recovery.

IMR is based on a review of controlled research (Mueser et al 2002a) in which five types of interventions (Mueser et al call them 'strategies') emerged which were incorporated into the training program.

These include psycho-education, cognitive behavioral approaches to improve medication compliance (medication management), relapse prevention by developing a crisis plan, strengthening social support with training in social skills and training to learn skills to cope with persistent symptoms (coping skills training).

To motivate patients to learn how to deal better with their disease and to make progress in their lives, IMR begins with an exploration of the meaning of recovery for the patient and the setting of personal recovery goals that can be worked on during the training course. The program includes 11 modules (see 6. Intervention).

It is intended that patients follow all modules. The total duration of the training program is on average one year, but the duration varies, depending on the frequency of sessions and the number of sessions per module. IMR may be offered individually or in a group format.

*The importance of recovery*
Anthony et al. (2003) introduced the term 'recovery' as a new and complementary approach to the rehabilitation process. According to Anthony, recovery includes much more than regaining mental health. The patients must also get over the stigma, the traumatic effects of certain practices, the lack of opportunities to shape their lives and the lost perspective on the future. According to Anthony the term means: "recovering the sense of self, again entering into a relationship with other people, converting powerlessness to power, giving new meaning to life and the retrieval of hope".

In the Netherlands the patient movement headed by Wilma Boevink has adopted the term "herstel" (recovery). Boevink (2002) says "recovery is something that patients do themselves. It is a search for how to deal with mental health problems, how to get a grip on the problems and how to put life increasingly in your own hands. Recovery is much more than cure."
Bond et al. (2005) noted that in the Netherlands there is too much emphasis on crisis intervention and stabilization of the patient. He advises investing in a recovery oriented approach. In his opinion, patients have more capabilities if they are well informed and facilitated. According to Bond elements of a recovery orientation are an active collaboration with the patient to reach his personal goals, the propagation of a consistent message of hope and optimism at each contact, giving priority to the choice of the patient in all aspects of the treatment, housing, work and medication, and emphasize the strength of the patient.
In their review of the research, Mueser et al. (2002a) say that recovery refers not only to relief from symptoms but also to social success and personal accomplishment in areas that the person defines as important. According to their paper recovery has been conceptualized as a process, as an outcome, and as both. What they call critical about recovery is the personal meaning that each individual attaches to the concept. Common themes of recovery, which they name, are the development of self-confidence, of a self-concept beyond the illness, and of a sense of well-being, hope, and optimism. Recovery has therefore to do with empowerment, perceived self-stigma and setting and achieving of meaningful goals.
Dröes & Plooy (2010) distinguish three different areas of recovery: recovery from illness, recovery of individual roles and recovery of personal identity. In the study by Bogaards et al (Bogaards et al. 2010, Oosterbaan et al. 2011) a fourth area is added: recovery of daily functioning.

*Illness Management*
Mueser et al. (2002a) define illness management as 'professional-based interventions designed to help people collaborate with professionals in the treatment of their mental illness, reduce their susceptibility to relapses, and cope more effectively with their symptoms'. Illness management means that the patient takes control over his illness and learns to intervene to prevent relapse. The patient learns too to (re) influence his social environment and to find his way to the appropriate care. The main goal of IMR is to offer people the right information and skills to allow them to make for themselves the right choices in their treatment. In this way IMR moves to meet the needs of the patient movement. The care provider tries to support the recovery process of the patient (together with his friends/family) and to help the patient to fulfill his role as a citizen.
Mueser et al. (2002a) call it critical to peoples' developing hope for the future to formulate personal recovery goals to help them to gain mastery over their symptoms and relapses. Basic education about mental illness facilitates their ability to regain control over their lives and to establish more collaborative and less hierarchical relationships with professionals.
The care professional takes on a less dominant position and the patient, and his or her peer group have space to work together with professionals to optimize the treatment.
This fits well with the ideas of Boevink (2002): "integration of Mental Health Care has no chance of success if only the services integrate into society".
Equally important is that the people who use these services change their identity of psychiatric patient to that of participants in society. " Mueser et al. (2002a) describe the advantages of IMR as: "Illness management is a broad set of strategies designed to help individuals with serious mental illness collaborate with professionals, reduce their susceptibility to the illness, and cope effectively with their symptoms. Recovery occurs when people with mental illness discover, or rediscover, their

strengths and abilities for pursuing personal goals and develop a sense of identity that allows them to grow beyond their mental illness”.
Mueser et al (2002a) say that illness management and recovery are closely related, with illness management focused primarily on minimizing people’s symptoms and relapses and recovery focused primarily on helping people develop and pursue their personal goals.

*Drop-out*
IMR is a relatively long-term care program (at least nine months at one session per week) for chronic psychiatric patients, usually involving starting of group sessions after completing the first individual module. Various publications and our own experience show that in different phases of the IMR program relatively high drop-out from treatment occurs. The overall drop-out in the IMR study of Hasson-Ohayon et al (2007) was 29% (60 out of 210; there is no indication of how this is split between the two research groups). The overall dropout rate in the study of Levitt et al (2009) (executed in a supported housing setting) was 15% (intervention group: 19%, control group: 12%). During the visit in 2009 of some staff members of BavoEuropoort to the U.S., it appeared that in Manchester (NH), there was 50% dropout from treatment of patients of ACT teams who participated in an IMR training. The drop-out from treatment in the pilot-study of Bavo Europoort from the moment of starting Module 1 was 44% (32 of 72, there were 40 completers, i.e. 56%).

### 1.2.2 Practical significance
This study aims at demonstrating the effectiveness of IMR on the illness management skills and recovery of the patients.
The added value for participants on different areas of life is examined. The research can contribute to answering the question whether IMR should be a recommended intervention.
Research can point to types of patients for whom IMR could be effective and conditions for success.

### 1.2.3 Scientific significance
The research may contribute to answering the question whether IMR can be called an EBP.
In the RCT by Hasson-Ohayon et al. (2007) IMR participants showed significant improvements in knowledge about their illness and showed progress towards their personal goals compared to patients with care as usual (CAU). Scores of clinicians showed a significant improvement in 'overall outcome'. Both the IMR- as the CAU group showed a significant improvement in coping and neither group showed change in social support.
In the RCT by Levitt et al (2009), executed in supported housing the intervention group scored significantly better in illness management and clearly had fewer symptoms and better psychosocial functioning.
In the RCT by Färdig et al (2011), the IMR-participants showed better illness management, less symptoms, better psychosocial functioning.
An effective factor in IMR in groups could be peer support (see Castelein et al, 2008a and 2008b and Castelein 2009), for which also indications come forward in our pilot study.
Nevertheless, in the field of psychiatric research in the Netherlands there still are doubts about the evidence-based status of IMR. There has not yet been done a RCT on IMR in the Netherlands. IMR is not yet mentioned in the multidisciplinary guidelines on Schizophrenia in the Netherlands (see 1.1).

*Transfer of knowledge*
IMR and the scientific status of IMR have a growing interest in the Netherlands and abroad. The results of the study are thus of significance for many people and will be studied by many.
The results of our study will be transferred by newsletters, conferences and articles in scientific journals.

## 2. Objective
The aim of this study is to examine the effectiveness of the IMR program compared to care as usual (CAU) in patients with SMI.

## 3. Research questions

Research questions are:
What are the effects of “IMR + CAU", compared to “CAU only" on:
1. Illness management, as experienced by clients (primary outcome measure)
2. Recovery.

The hypotheses are that "IMR + CAU", (IMR offered in group format), compared to “CAU only” leads to:

1. Better Illness Management:
(Better scores on IMR-scales, less symptoms and relapses, better medication adherence, less alcohol & drugs use, more insight into their own problems, more social and coping skills, more social support).

2. Better recovery:
(Better general recovery, less self-perceived stigma, more self-esteem, achievement of more meaningful goals, more quality of life, more satisfaction, and better social functioning).

3. Improved cost-effectiveness.

## 4. Design

The design is a randomized controlled trial in which patients are assigned to the experimental condition (IMR) or the control group, after providing written informed consent:

- Group 1. IMR program, offered in a group format + care-as-usual (CAU).
- Group 2. Care-as-usual (CAU)

At randomization, we apply the 'minimization method' (Beller et al 2002). This is a dynamic random allocation method, with the ability to stratify on variables that the outcomes (possibly) influence. We stratify on diagnosis (psychotic disorder yes or no) .
The procedure is performed using www.randomizer.org. Using the block-design we ensure that patients end up in the desired ratios in the two conditions (IMR group + CAU vs. CAU).

We have planned three moments of measurement. These moments are

1. prior to the randomization
2. after the training (in the experimental condition this time may vary depending on the duration of the group, the mean duration of the training in the pilot study was 12.6 months).
   The final (second) moment of measurement for the control group is 12 months after the first moment of measurement.
3. The follow-up measurement is 6 months after the second measurement.

## 5. Study population

### 5.1 Patients

Inclusion criteria are:

- Patients with serious and persistent psychiatric illnesses. Most of them will be patients who have a psychotic disorder, schizoaffective disorders or bipolar disorders with or without comorbid disorders (such as substance abuse and personality disorders)
- The patient is treated on an outpatient basis
- Written informed consent .

### 5.2 Exclusion criteria

Exclusion criteria are:
- Having done an IMR-training
- Organic brain syndrome.
- Incompetence regarding the giving of informed consent.
- Patients with severe cognitive impairments who are unable to follow the training
- Insufficient knowledge of the Dutch language (so that they cannot participate in the group).

### 5.3 Sample Size

Based on the effect sizes of the studies of Hasson et al. (2007) with 210 participants (.41, Cohen's d), Levitt et al. (2009) with 104 participants (.36) and Färdig et al. (2011) with 41 participants (.60) we expect a medium effect size of 0.40.
Based on the power analyses with three moments of measurement (mixed models) and equal allocation to experimental and control group, a power of .80, correlation between measures .50, alpha at 0.05 and an effect size of .40, we need to randomize 148 patients, 74 to the experimental condition and 74 to the CAU-group (see Twisk (2003) and Guanghan L. and Kung-Yee L. (1997).

Because of the (conservatively estimated) expected drop-out from treatment in the experimental condition of 50% we choose to allocate more patients to the experimental (IMR-) condition than to the CAU-group (proportions of 3:2). Then we need to randomize 185 patients, 111 patients to the experimental condition and 74 to the CAU-group.
Because we expect a total drop-out in each group from measurements of 8 % we need 80 patients in the control condition and 120 patients in the experimental condition. In total: 200 patients (see Annex 1).

## 6. Intervention

The average duration of the total IMR-training is 12 months. The IMR-training consists of 11 modules that are given weekly. The first module is given individually. During this individual module the patients decide which goals they want to work on during the program.
Then the patients join an IMR group for the other modules. Each module takes about 3 to 4 sessions of one and a half hours each. The IMR group is guided by two trainers (psychiatric nurses). The trainers received a two-day course in IMR and attend supervision once every two weeks.

The modules are described in the IMR-workbooks, translated into Dutch, which the patients received. If necessary, the original American text is adapted to the Dutch context. The modules are: 1. Recovery Strategies, 2. Practical Facts about Mental Illness, 3. Stress-Vulnerability Model, 4. Building Social Support, 5. Using Medication Effectively, 6. Alcohol and Drugs Use, 7. Reducing Relapses, 8. Coping with Stress , 9. Coping with Problems and Persistent Symptoms, 10. Getting Your Needs Met in the Mental Health System, 11. Health for you.

The trainers use techniques from motivational interviewing, psychoeducation and cognitive-behavioral therapy (CBT). Peer group support is part of the IMR-training. Home assignments are provided. Workbooks and homeassignments can be accessed via the internet. There is feedback on homework from trainers on the Internet. A patient is considered to be a ''completer'' of the IMR-training if he has completed at least 70% of the sessions.

The quality of the implementation of the intervention is checked in two ways:

1. Degree of fidelity of the implementation of IMR. (Quality of implementation).
To measure whether the program is implemented according to the original intent, the researchers determine the model fidelity using the IMR fidelity scale (Mueser et al. 2002b).
A translation of this scale has been made and training in the use of the IMR fidelity scale has been followed in collaboration with M. Salyers, Ph.D. and A. Mc Guire, Ph.D of UIPUI, University of Indianapolis USA, together with Saxion Academy Deventer.

2. Quality of the trainers.
To be able to measure this, we will use a scale recently developed by UIPUI in Indianapolis USA: the Illness Management and Recovery Treatment Integrity Scale (IT IS). This scale rates the quality of the trainers with respect to having experience in guiding groups, (social) skills training, ability to structure role playing and to integrate text of the workbooks with personal goals and problems of a participant, and maintenance of a positive attitude. Publications are submitted.

## 7. Intervention- and control group

Group 1. Experimental condition
These patients participate in the IMR-training as well as CAU.

Group 2. Control Condition
These patients only get CAU. This consists of regular individual meetings (usually every two weeks) with a community psychiatric nurse (in Dutch: SPV). The topics are related to symptoms and handicaps related to the illness and methods for managing them. The applied methods include eg supportive therapy, CBT or help in finding (volunteer) work.
If CBT is indicated it can be given by a psychologist or a behavior therapy assistant. The SPV may also help to solve practical problems such as finding housing or the cleaning up of debts.
At least once a year, the patient has an appointment with a psychiatrist for the optimization of medication and a somatic examination.

The difference between the two conditions is that the patient in the 'IMR + CAU' condition follows a structured IMR-training, aimed at achieving their own goals and learning of 'illness management'. We expect that patients less systematically work on their personal goals during 'CAU only’ and learn less about illness management. To control for type of interventions in CAU, all interventions will be registered.

## 8 Methods

### 8.1 Outcome measures

#### 8.1.1 Primary outcome measures

- Illness management and recovery scale-patient self score version

There are two identical IMR-scales (Mueser et al 2004; Salyers et al 2007; Hasson-Ohayon et al. 2008; Dutch translation De Vries 2011) with 15 items completed by patients themselves and by the clinicians, respectively. The patient version of the IMR-scale will be the primary outcome measure.

The consumer and clinician versions of the Illness Management and Recovery (IMR) scales have adequate psychometric properties. These scales have good internal consistency and high 2-week test-retest reliability. Both versions had good convergent validity (Salyers et al. 2007).
Factor analysis of both scales showed three similar client and clinician factors: knowledge and goals, coping with illness and a factor that combined effective medication use and reduced alcohol and medication use. These factors explained 40% of the total item variance for the client scale and

47% of the total item variance for the clinician scale. These factors are found to have moderate reliabilities. (Hasson-Ohayon et al. 2007).
The scales have been translated into Dutch and then independently back-translated into English and compared with the original version to identify and correct discrepancies.

### 8.1.2. Secondary outcome measure

#### a. Illness management

**IMR-scale clinician-rated version will** be used to explore effects. This scale is scored by non-blinded clinicians, who are not involved in the IMR-training.

Since the IMR-scales measure multiple dimensions in which each dimension is covered by only one or two questions, some extra questionnaires will be used to further explore & validate the concept of illness management and recovery. These scales are chosen based on the content of the concept of IMR. For this purpose we disentangled the IMR concept into different domains related to illness management and recovery, respectively

**Coping:** Coping self-efficacy scale (CSES, Chesney et al. 2006) (13-items):
**"**Exploratory factor analyses (EFA) and confirmatory factor analyses (CFA) revealed a 13-item reduced form of the CSE scale with three factors: Use problem-focused coping (6 items, $\alpha = 0.91$), stop unpleasant emotions and thoughts (4 items, $\alpha = 0.91$), and get support from friends and family (3 items, $\alpha = 0.80$). Internal consistency and test–retest reliability are strong for all three factors. Concurrent validity analyses showed these factors assess self-efficacy for different types of coping. Predictive validity analyses showed that residual change scores in using problem- and emotion focused coping skills were predictive of reduced psychological distress and increased psychological well-being over time". The CSE scale provides according to Chesney et al. a measure of a person's perceived ability to cope effectively with life challenges, as well as a way to assess changes in CSE over time in intervention research.

**Social support:** Multidimensional Scale of Perceived Social Support MSPSS (Zimet et al.1988) (12 items). The MSPSS was found to have good internal reliability across subject groups. In addition, strong factorial validity was demonstrated, confirming the three-subscale structure of the MSPSS: Family, Friends, and Significant Other. Strong support was also found for the validity of the Family and Significant Other subscales.

**Medication compliance:** Service Engagement Scale (SES) (14-items)
The Service Engagement Scale (Tait et al. 2002) is a 14-item measure consisting of statements that assess client engagement with services, which case managers rate on a four-point Likert scale from 'not at all or rarely' to 'most of the time'. The total score ranges from a minimum of zero to a maximum of 42. Higher scores indicate lower engagement. Four sub-scales assess availability ('when a visit is arranged, the client is available'), collaboration ('the client actively participates in managing his/her illness'), help-seeking ('The client seeks help to prevent a crisis') and treatment adherence ('The client refuses to cooperate with treatment'). The scale has high internal consistency and retest reliability, including discrimination between criterion groups, in an assertive outreach team (Tait et al, 2002).

**Insight** into own problems: Insight Scale (IS, 8 self-report items) (PI-scale) (Birchwood et al, 1994). This 8-item self-report scale was designed to be sensitive to changes in levels of insight, and captures three dimensions of insight: perceived need for treatment, awareness of illness and relabeling of symptoms as pathological. Higher scores indicate greater levels of insight. The psychometric properties of the scale are called excellent (Tait et al. 2003).

**Symptoms:** The Brief Symptom Inventory (BSI) (53 items) (Derogatis & Melisaratos 1983; Derogatis 1993; De Beurs, 2008). The BSI instrument (self-report scale) provides an overview of a patient's symptoms and their intensity at a specific point in time and can be used to measure patient progress during and after treatment to monitor change. The BSI test is brief and requires 8-10 minutes to complete, making it well-suited for repeated administrations over time to evaluate patient progress. The authors report good internal consistency reliability for the nine dimensions , ranging from .71 on Psychoticism to .85 on Depression. Test-retest reliability for the nine symptomdimensions ranges from .68 (Somatization) to .91 (Phobic Anxiety), and for the three Global Indices from .87 (PSDI) to .90 (GSI). Studies attesting to the validity of the BSI are found in the manual (Derogatis, 1993).

**Relapses:** The number of relapses (operationalized in the number of hospital admissions) during and after participating in the IMR-training will be compared with the number of relapses in the year before participating in IMR.

**Alcohol & Drugs:** One item (item 24) of the Addiction Severity Index (ASI) (McLellan et al, 1980,. Hendriks et al, 1989), asking how much respondents has been bothered the past 30 days by problems with a. alcohol, b. drugs, (a & b separately scored on a 5-point scale).

**b. Recovery**

The concept of recovery is complex. We choose to assess recovery by using a special scale as well as measuring different aspects of recovery including aspects of what Mueser (see 1.1) calls subjective recovery (self esteem, self stigma, quality of life, satisfaction) and objective recovery (functioning). The outcomes of the intervention on these variables and the association with the outcomes on the primary outcome measure will be tested on an exploratory basis.

- **Recovery**: The Mental Health Recovery Measure (MHRM) (Young & Bullock, 2003), Bullock, 2005); authorized translation in Dutch (Moradi et al, 2007) (30 items).
The MHRM is a 30 item self-report measure designed to assess the recovery process for individuals who have serious and persistent mental illnesses such as recurrent major depression, bipolar disorder, or schizophrenia spectrum disorders. The MHRM is scored using a 5 point Likert Scale (0 to 4) for each item, yielding a theoretical range from 0 – 120 for Total Score. The internal reliability (coefficient alpha) of the MHRM Total Score was .93. One-week test-retest reliability was .92. This scale has three subscales: self-empowerment (13 items), learning & new-potentials (15 items), spirituality (2 items).
According to Mc Cabe et al. (2007) the MHRM assesses three phases of recovery: overcoming sickness; discovering and fostering self-empowerment; and striving to attain overall well-being and reach new potentials.

**- Goals:** Granholm's Goals Template measures progress towards goals on 10 life domains (Employment, Housing, relationships, school, self-care; leisure activities, addictions, money management goal, independence using transportation, general template) and is a method of obtaining objectivation of progress towards personal goals.

- **Self stigma**: The Internal Stigma of Mental Illness (Ismi), 29 items.
This scale is designed to measure the subjective experience of stigma, with subscales measuring Alienation, Stereotype Endorsement, Perceived Discrimination, Social Withdrawal and Stigma Resistance. The ISMI has 29 Likert items. The ISMI has high internal consistency and test–retest reliability. Construct validity and divergent validity was supported by comparisons against scales measuring related constructs with the same methodology. The ISMI has positive correlations with measures of stigma beliefs and depressive symptoms, and negative correlations with measures of self-esteem, empowerment and recovery orientation. Factor analyses of the joint set of items from the ISMI and each scale supported the distinction between constructs (Ritsher 2003).

**- Self esteem:** The Self-Esteem Rating Scale-Short Form (SERS-SF), 20 items.
This scale of 20 items (the original scale has 40 items) has two subscales (positive and negative self-esteem), which is supported by factor analysis. Each subscale ranges from 10 to 70, higher scores indicate higher positive or higher negative self-esteem. This scale has good internal consistency, good test-retest reliability and adequate convergent validity in patients with schizophrenia. (Lecomte et al., 2006).

- **Quality of life:** The EQ-5D (Prieto et al 2003), 5 items.
The EQ-5D is the Euro-QOL self-report scale with 5 dimensions and good psychometric properties. According to a Dutch validation study by Lamers et al. (2005), Staring (2010) calculated the items into a weighed total score ranging from -0.33 to 1.00. Higher scores reflect better quality of life.

- **Satisfaction**, Two questions: ‘’Can you tell me how satisfied you are with your life as a whole? and “How satisfied are you with the health care services you visited?” both scored on a 7-point scale. These questions are used in the Routine Outcome Monitoring of the Long Stay sector of Parnassia Bavo Group and is supposed to correlate with all other possible satisfaction-questions which were part of satisfaction questionnaires. (see Delespaul et al. 2006)

- **Social functioning.** The Social Functioning Scale. 7 dimensions (Social withdrawal, relationships, social activities, recreational activities, independence (completence), independence (performance), employment.19 items and 4 checklists with in total 62 aspects. This Scale is called reliable, valid, sensitive and responsive to change (Birchwood et al. 1990).

**c. Cost-effectiveness**

**Cost-effectiveness**: The number and duration of contacts (including the IMR-meetings), crisis contacts, (forced) admissions and duration of admissions are calculated in costs in euro’s. These are related with changes in quality of life measured by the EQ-5D (see 8.1.2 c). By transforming scores on the EQ-5D in so called ‘Qualy’s’ cost-effectiveness can be calculated.
To calculate cost-effectiveness, only cost of health care consumption is included: no social costs such as rent, benefits, etc. The major costs are the costs associated with hospital admissions.

**8.1.3. Exploratory analysis**
The pilot study showed that women significantly more often are IMR completers and that completers at baseline scored better on the IMR-scales and on the GAF (psychosocial functioning). This makes it interesting to examine whether women benefit more from IMR than men and whether patients who at baseline score higher in terms of illness management, psychosocial functioning and symptoms, improve more than patients who at baseline score lower on these domains.
Earlier IMR research was often focused on patients with psychotic disorders. We want to investigate whether the effect varies by diagnosis group. We are able to explore this because in the IMR-training of Bavo Europoort chronic patients with various diagnoses participate.
Since half of the population of outpatients of Bavo Europoort has an immigrant background, we would like to explore whether IMR for participants from ethnic minorities is as effective as for participants of native background.

**8.2 Procedures**

**8.2.1 Selection of patients**
It is assumed that we need to screen approximately 687 patients for participation in the study. An estimated 515 patients (80%) will meet all inclusion criteria and will be asked by the clinicians to participate in the study. It is expected that about 40% of these patients are willing to participate in the study and then we get the amount we need (n = 200). Patients will be randomized in a ratio of 3:2. After drawing lots there will be 120 patients in the IMR-condition and 80 in the control condition.

We expect that in the IMR condition 37% will drop-out of IMR in the phase between randomisation and the inflow into the (individual) Module 1 and in the phase from module 1 to the inflow in the IMR group (module 2). We expect that in the IMR-condition there will approximately 78 patients who start the IMR- training groups. Based on the results of the pilot study it is expected that 77 % (n = 60) of these people will complete the IMR groups.
Drop out of the IMR-program does not mean dropping out of the study (see in 8.2.4 the intention-to-treat principle). We expect that of the 120 patients in the experimental condition, that in the second moment of measurement score at least 115 patients can be measured, and at follow-up measurements at least 111 patients. We expect that of the 80 patients in the control condition that in the second moment of measurement score at least 77 patients can be measured and at follow-up measurements at least 74 patients (See Annex 1, flowchart of randomisation). We expect this drop-out of the study is only 8% because patients who want to withdraw from care will not wish to participate in IMR.

**8.2.2 Training of clinicians**
Clinicians treating patients with severe and persistent psychiatric illnesses of BavoEuropoort will be involved in the research.
IMR group leaders have received an IMR-group leader-training of two days about the main aspects of IMR given by two experienced instructors of BavoEuropoort and they will all receive two-weekly supervision.

**8.2.3 Recruitment of patients**
First, the investigator will provide information to the team leaders of the aforementioned outpatient teams and ask for their participation in the study.
Three months before starting the randomization the clinicians of the six participating teams will receive the information about the study from the researcher followed by information on the process of randomization. During a treatment team meeting, the investigator will explain the research plan. Subsequently, the files of the aforementioned patients will be screened for the inclusion criteria.

After screening the files on the inclusion criteria potential participants will receive an information letter from their clinician and will be asked whether they accept that the researcher contacts them. The researcher will then contact the patient, explain the research project and ask for written informed consent.
If a patient refuses to participate he/she will be asked for permission to use demographic and diagnostic data in order to determine the generalizability of the sample. After signing the informed consent, the first measurement will take place, followed by the randomization.

**8.2.4. Research data**

Data on primary, secondary and tertiary outcome measures and the mediating factors will be collected at baseline (before randomization), after the IMR-training (from 9 to 12 months) and during follow-up (after 15 - 18 months ). The interviewers will be blind for the (experimental or control) condition of the patient. For the recruitment of participants and data collection, students will be employed.

During the first contact (at baseline) and after the patient has given informed consent, the research interview will take place and the interviews will be conducted. Subsequently randomization will take place. Each assessment will last up to 80 minutes. If desired, the interview will take place on two occasions. The assessments will be done at the location where the patient usually comes.
Besides the previously mentioned outcome measures and mediating factors at baseline the following data will also be collected:
- Demographic data: age, sex, living situation, source of income, education level, country of birth of the patient and of his father and his mother.

- Consumption of mental health care: number of outpatient contacts, number of crisis contacts, number of admissions and the number of compulsory admission orders (total and in the last two years) during the past two years before and after moment of measurement 1.
- Psychiatric diagnosis: DSM-IV-TR diagnosis (five axes) according to the clinician.
We realize that this is not always reliable, but it is not possible with the resources available to do a standardized diagnostic interview.

The analysis will be based on the intention to treat principle. Data will be collected of patients who drop out of the IMR program. Based on the pilot study, during the IMR intervention, 50 % drop out from IMR is expected. In order to do analyses related to generalizability of the study, the demographic characteristics of all patients who refused to participate will be registered, as well as the characteristics of the patients who drop-out from the study.

The demographic variables, consumption of mental health care and psychiatric illness will be collected by the researcher from the administration of the institution, supplemented by data from the file.
The researcher can not be blind for the (experimental or control) condition of the patient.
Number, duration and nature of all treatments which people get in both conditions are recorded. We expect that in the "IMR + CAU"-condition the patient will use less CAU than in the' CAU only'-condition.
In addition, we will examine exploratory the mechanisms behind a (possible) effect of IMR as well as the experiences with IMR in practice.

The application of the questionnaires may last in total one hour. At the first interview patients receive an amount of € 20, -, at the moment of measurement of the final-scores € 25, and at follow-up measurements € 30, - as a sign of appreciation for participation in the study.

The research data will be stored and reported in an anonymous manner. Personal data are coded. The research team has access to the key of the code.

### 8.2.5. Organization of data collection

- The students who are employed for the recruitment of participants and the data collection are trained by the researcher.
- There is a budget calculated (see Annex 2). Each interview of one patient will last on average one hour, considering that all questionnaires together have 273 items. You could do two interviews in half a day, but you need a lot of preparation and aftercare for each visit.

### 8.4 Participating sites

The following IMR-teams (teams for outpatient care)/ locations within BavoEuropoort will participate in the study:

IMR Team Noord Oudedijk (Rotterdam)
IMR Team, Oost Oudedijk (Rotterdam)
Time-out Team Carnissesingel (Rotterdam)
IMR Team Zuid Carnissesingel (Rotterdam)
IMR Team Centrum Westblaak (Rotterdam)
IMR Team Zuid-Hollandse Eilanden (Spijkenisse).

## 9 Statistics

- For measurement of the effects of the IMR-training on the primary outcome measure, and on secondary outcomes on three moments of measurement we use Linear Mixed Models.

Differences in time between groups can be examined with cross level interaction for time x group interaction effect.

- The groups are compared at the final and follow-up assessments in terms of a main group effect, to test the question whether the groups differ after treatment, controlling for baseline.

- For validation of the scores on the primary outcome measure on different aspects of Illness Management (Stress, Social Support, Medication adherence, Drugs use, Symptoms/Remission/ Relapse, Coping) we use multiple regression analysis, logistic regression analysis and multinomial analysis.

‘For prediction of the effect on the primary outcome measure using the baseline data in terms of functioning (scores on IMR-scales, GAF-scores, Social Functioning Scale and BSI) we use linear regression analysis.
For prediction of the effect on the primary outcome measure using the baseline data in terms of patient characteristics (gender, etnicity, diagnosis) we use t-tests and one way ANOVA.’

– If we find effects of the IMR-training on the Distal outcome measures of recovery:
to determine the influence of Illness management aspects as mediating factors on the effects on recovery we use Multiple Mediator Analysis for SPSS (Preacher & Hayes, 2008).

## 10 Feasibility of the study

The organization of the IMR training and the organization and logistics of the research require a major effort by Bavo Europoort and the research team.
Given the successful progress of the pilot study it seems that the RCT-study is quite feasible.
IMR has become a familiar type of care in the outpatient teams and there is enthusiasm among clinicians and patients. Moreover, there is now an infrastructure for training and supervision of the IMR trainers. Data collection in the pilot study went relatively smoothly. It is difficult to predict the effect of the randomization procedure on the willingness of the patients to participate in the study.

A patient who accepts participation in the control group is essentially ineligible for IMR for a period of 1.5 years. Earlier research showed that about 50% of patients are willing to participate in such an investigation (Henderson et al 2004). Participation often appears very dependent on how clinicians and researchers approach the patient.
For the 85 patients who supposedly will be in an IMR-group in the experimental condition 10 IMR groups need to start in the first year of data collection (baseline measurement). This requires 20 trainers.
The 6 IMR-teams of Bavo Europoort have approximately 60 employees. Everyone is trained in IMR. It is therefore theoretically feasible to undertake the study, and would also make IMR a more explicit component of the care provisions of the group. However, to implement IMR on such a broad scale and to participate in the study is still a challenge for the institution.
We fully anticipate that the enthusiasm of clinicians for this study will lead to the inclusion of the required patient numbers for the study.

## 11 Time frame

The planning is based on a period of 1 year in which 14 IMR-groups will start and the baseline measurements of all patients will be obtained.
The duration of the 6-IMR groups in the pilot was on average 12.6 months. The following schedule has been assumed that the duration of the groups is 12 months.

Start: April 1th 2011

0 - 15 months ( July 2012). Writing definitive protocol, asking permission of medical ethical committee, request of additional grant, training in IMR fidelity-scale, informing clinicians, management, recruitment of students.

15 months (July 2012)- 29 months ( September 2013)., training of students, patient recruitment, baseline measurements

17 months (Sept 2012) - 41 months (September 2014). IMR program is offered.

29 months (Sept 2013) - 41 months (September 2014) Second measurements

35 months (February 2014) - 47 months (March 2015). Follow-up measurements

47 months (February 2015) - 60 months (June 2016). Entering data into the computer, analysis and reporting of results. Processing of data in articles and a dissertation

The expected completion date of the study is July 1th, 2016.

## 12 Ethical considerations

### 12.1 Recruitment and informed consent

It seems more familiar for the patient to hear about the investigation through the therapist. Explanation of the study, however, is given by the investigator and not by their own therapist. This will guarantee the freedom of choice of the patient. The researcher stresses the voluntariness of participation of the patient and that refusal of participation, will cause no change on its own treatment. Patients can only participate in the study if they sign an informed consent form.

### 12.2 Randomization and refusal

The effects of IMR are not yet known. Therefore we think it is ethical to allocate patients randomly to two conditions. After 15-18 months, patients in the CAU group can start IMR.
Patients who refuse to participate in the randomization, can still express their preference for one of the two conditions, bur are excluded from the study. This means that they can participate in IMR if they do not wish to participate in the study.

## 13 Funding

The total budget of the investigation is € 337.830,- (see Annex 2). The researcher is funded by BavoEuropoort for five years for 2.5 days per week and for five years for one half day per week by the Parnassia Bavo Academy (In total € 250.000,-). An unconditioned grant of € 87.830,- for additional costs has been obtained by BavoEuropoort from Janssen-Cilag B.V.

***Planned papers***

- BMC Article
- English Article about the pilot-study
- Leading article concerning the effectiveness of IMR
- Article on qualitative aspects
- 2 Articles (cross sectional over the baseline data)
- Dutch article about the pilot, if we have time.

### *Literature*

Anthony, WA, Rogers, ES & Farkas, MD (2003). Research on evidence based practices: Future directions in an area of recovery. Community Mental Health Journal, 39, 101-114.

Beller EM, Gebski V, Keech AC. Beller EM, Gebski V, Keech AC. Randomisation in clinical trials. Medical Journal of Australia 2002, 177:565-567.

Birchwood M., Smith J, Cochrane R, Wetton S and Copestake S (1990). The Social Functioning Scale. The development and validation of a new scale of social adjustment for use in family intervention programmes with schizophrenic patients. British Journal of Psychiatry, 157:853-859.

Birchwood,M., Smith, J., Drury,V., et al (1994) A self-report insight scale for psychosis: reliability, validity and sensitivity to change. Acta Psychiatrica Scandinavica, 89, 62-67.

Boevink W. (2002), Samenwerken aan herstel, van ervaringen delen naar kennis overdragen, Trimbos-instituut, Utrecht. (Collaborating on recovery, from sharing of experiences to the transfer of knowledge, Trimbos Institute, Utrecht)

Bogaards M., H. Oosterbaan en B.J. Roosenschoon (2010), Herstel in een rehabilitatiecentrum, verslag van een onderzoek onder cliënten van het Rehabilitatiecentrum van Bavo Europoort, Onderzoek & Ontwikkeling Bavo Europoort, Rotterdam.
(Recovery in a rehabilitation center, report of a survey on patiënts of the Rehabilitation Center of Bavo Europoort Rotterdam, Bavo Europoort, Research & Development).

Bond G.R., ea (2005), Toekomstige ontwikkelingen van ACT. (Future developments on ACT). In: CL Mulder & H. Kroon (red.) Assertive community treatment, Nijmegen, Cure & Care publishers.

Bovenberg, F., Hiwat, M. & Roosenschoon, B.J. (2006), Ziektemanagement en herstel: eerste ervaringen met de implementatie van Illness Management & Recovery. (First experiences with the implementation of Illness Management & Recovery). Passage, 15, 26-34.

Bovenberg, F. and H. Staats (2008), Illness Management & Recovery. In: Plooy A. ea (red.), Pychiatrische rehabilitatie, Jaarboek 2008-2009, SWP Amsterdam.
(In: Plooy A. et al (eds.), Psychiatric rehabilitation, Yearbook 2008-2009, SWP Amsterdam.)

Bullock, W. A. (2005). The Mental Health Recovery Measure. In Campbell-Orde, T., Chamberlin, J. Carpenter, J., & Leff, H. S. (Eds.) Measuring the Promise of Recovery: A Compendium of Recovery Measures. Volume II. The Evaluation Center@HSRI: Cambridge, MA.

Castelein, S., Bruggeman, R., van Busschbach, JT, van der Gaag, M., Stant, AD, Knegtering, H. et al (2008a). The effectiveness of peer support groups in psychosis: a randomized controlled trial. Acta Psychiatrica Scandinavica, 118, 64-72.

Castelein, S., Mulder, PJ, & Bruggeman, R. Castelein, S., Mulder, PJ, & Bruggeman, R. (2008b). Guided peer support groups for schizophrenia: A nursing intervention. Psychiatric Services, 59, 326.

Castelein S. (2009), Guided Peer Support Groups for Psychosis, A randomized controlled trial, Academisch Proefschrift RU Groningen. (Guided Peer Support Groups for Psychosis, A randomized controlled trial, Academic Thesis RUGroningen).

Chesney, M. A., Neilands, T. B., Chambers, D. B., Taylor, J. M. and Folkman, S. (2006), A validity and reliability study of the coping self-efficacy scale. British Journal of Health Psychology, 11: 421–437. doi: 10.1348/135910705X53155

Corrigan PW, McCracken SG, Holmes EP (2001). Motivational interviews as goal assessment for persons with psychiatric disability. Community Mental Health Journal;37:113–122.

Cosway R., Endler  N. S., Sadler A.J And Deary I.J. (2000), The Coping Inventory for Stressful Situations: Factorial Structure and Associations With Personality Traits and Psychological Health. Journal of Applied Biobehavioral Research, 5, 2, pp. 121-143.

De Beurs, E. (2008). Brief symptom inventory handleiding. Leiden: The Netherlands: PITS B.V. Delespaul, Ph. A.E.G., Bak, M.L.F.J. & van Os, J. (2006). Handleiding Zorgmonitor 2006. (5de Uitgave) Maastricht: Universiteit Maastricht.

Derogatis, L.R. & Melisaratos, N. (1983). The Brief Symptom Inventory: an introductory report. Psychological Medicine, 13(3), 595-605.

Derogatis, L. R. (1993). BSI Brief Symptom Inventory. Administration, Scoring, and Procedures Manual (4th Ed.). Minneapolis, MN: National Computer Systems.

Drake, RE, Goldman, HH, Leff, HS, Lehman, AF, Dixon, L., Mueser, KT, et al. (2001). Implementing evidence-based practices in routine mental health service settings. Psychiatric Services, 52(2), 179–182.

Droes JM en Annette Plooy (2010), Herstelondersteunende zorg in Nederland: vergelijking met Engelstalige literatuur, Tijdschrift voor Rehabilitatie, 19, (2), 6-16. (Recovery Supportive care in the Netherlands: comparison with English literature, Dutch Journal of Rehabilitation, 19, (2), 6-16. )

Ebbers, L. (2008) Implementatieplan IMR, Rotterdam Bavo Europoort.

Färdig R., T.Lewander, L.Melin, F.Folke, A.Frederiksson (2011), A randomized Controlled Trial of the Illness Management and Recovery Program for Persons with Schizophrenia. Psychiatric Services 62: 606–612.

Gaag M. van der (2008) , Illness Management & Recovery, Impossible Mission Revisited, Presentation Heiloo, the Netherlands.

Granholm E. (2011), Granholm's Goal Templates CBSTS, personal communication.

Guanghan L. and Kung-Yee L. (1997), Sample Size Calculations for Studies with Correlated Observations, Biometrics, Vol. 53, 3 , pp. 937-947.

Hendriks V; Kaplan C; Van Limbeek J; Geerlings P (1989). The Addiction Severity Index: Reliability and validity in a Dutch addict population. Journal of Substance Abuse Treatment, 6: 133- 141.

Hasson-Ohayon, I., Roe, d., Kravetz, S. (2007). A RCT of the effectiveness of IMR. Psychiatric Services, 58, 1461-1466.

Hasson-Ohayon, I., Roe D., Kravetz S. (2008), The psychometric properties of the illness management and recovery scale: Client and clinician versions, Psychiatry Research, 160, 228–235.

Henderson C, Flood C, Leese M, Thornicroft G, Sutherby K, Szmukler G. (2004). Effect of joint crisis plans on use of compulsory treatment in psychiatry: single blind randomized controlled trial. BMJ; 329:136-138.

Lamers LM, Stalmeier PFM, McDonnell J, Krabbe PFM, Van Busschbach JJ. Measuring the quality of life in cost-utility analyses: the Dutch EQ-5D tariff. Ned Tijdschr Geneesk. 2005;149:1574

Lecomte T., Corbière M., Laisne F. (2006), Investigating self-esteem in individuals with schizophrenia: Relevance of the Self-Esteem Rating Scale-Short Form Psychiatry Research 143 99–108

Levitt, A., Mueser, K., DeGenova, J., Lorenzo, J., Bradford, D., Barbosa, A., Karlin, M., Chernick, M. (2009). A RCT of IMR in multi-unit supportive housing. Psychiatric Services 60:1629-1636.

Liberman RP, Mueser KT, Wallace CJ, Jacobs HE, Eckman T, Massel HK. Training skills in the psychiatrically disabled: learning coping and competence. Schizophr Bull. 1986;12: 631–647.

McCabe R., Saidi M. and Priebe S. (2007), Patient-reported outcomes in schizophrenia, British Journal of Psychiatry, 191 (suppl. 50), s21-s28 . doi : 10.1192 / bjp.191.50.s21

McLellan AT; Luborsky L; Woody GE; O'Brien CP (1980). An improved diagnostic evaluation instrument for substance abuse patients: the Addiction Severity Index. Journal of Nervous and Mental Disease, 168: 26-33.

Miles, MB & Huberman, AM (1994). Qualitative data analysis (2nd ed). Thousand Oaks, CA: Sage.

Miller WR, Rollnick S, eds. Motivational Interviewing: Preparing People for Change. New York, NY: Guilford Press; 2002.

Mueser KT, Corrigan PW, Hilton D, et al. (2002a), Illness Management and Recovery: A Review of the Research, Psychiatric Services 53:1272-1284.

Moradi, Brouwers, Van den Bogaard & Van Nieuwenhuizen, 2007, authorized translation in Dutch of the Mental Health Recovery Measure (MHRM), University of Tilburg/ Tranzo.

Mueser K., Gingerich S., Bond G., et al. (2002b). Illness Management and Recovery Fidelity Scale, in Illness Management and Recovery Implementation Resource Kit. Edited by Mueser K., Gingerich S. Rockville, Md, Substance Abuse and Mental Health Services Administration.

Mueser K.T. et al. (2003), Implementing Evidence-Based Practices for People With Severe Mental Illness. Behavior Modification, 27 (3), 387-411

Mueser, KT, Meyer, PS, Penn, DL ea (2006). The illness management and recovery program: rationale, development, and preliminary findings. Schizophrenia Bulletin, 32, S32-S43.

Mulder, C.L., Staring, A.B.P., Loos, Buwalda, V.J.A., Kuijpers, D., Sytema, S.& Wierdsma, A.I. (2004), De Health of Nation Outcome Scales (HoNOS) als instrument voor 'routine outcome assessment'. Tijdschrift voor psychiatrie, 46 (5): 273-284

Oosterbaan H., M., M. Bogaards, BJ Roosenschoon (2011), Cliënten over herstel; onderzoek in een Rehabilitatiecentrum. Maandblad Geestelijke Volksgezondheid, 66 (3) 147-160. (Patiënts on recovery, research in a Rehabcenter)

Preacher, K. J., & Hayes, A. F. (2008). Asymptotic and resampling strategies for assessing and comparing indirect effects in multiple mediator models. *Behavior Research Methods*, *40*(3), 879-891. doi:10.3758/BRM.40.3.879

Prieto, L., Novick, D., Sacristán, JA, Edgell, ET, Alonso, J. and on behalf of the SOHO Study Group (2003), A Rasch model analysis to test the cross-cultural validity of the EuroQoL-5D in the Schizophrenia Outpatient Health Outcomes Study. Acta Psychiatrica Scandinavica, 107: 24–29. doi: 10.1034/j.1600-0447.107.s416.6.x

Prochaska JO, DiClemente CC. The Transtheoretical Approach: Crossing the Traditional Boundaries of Therapy. Homewood, Ill: Dow-Jones/Irwin; 1984.

Prochaska JO. Systems of Psychotherapy: A Transtheoretical Analysis. HomewoodIll Dorsey; 1984.

Ritsher J.B., Otilingam P.G., Grajales M. (2003), Internalized stigma of mental illness: psychometric properties of a new measure, Psychiatry Research 121 31–49.

Salyers, MP, JL Godfrey, KT Mueser, S. Labriola (2007) Measuring Illness Management Outcomes: A Psychometric Study of Clinician and Consumer Rating Scales for Illness Self Management and Recovery. Comm. Mental Health Journal, 43, 5.

Staring A.B.P. (2010), 'Adherence to Treatment in Patients with Psychosis'. Academic Thesis, Erasmus University Rotterdam.

Tait L., Birchwood M. and Trower P., Predicting engagement with services for psychosis: insight, symptoms and recovery style. The British Journal of Psychiatry 2003 182: 123-128.

Twisk, J.W.R. (2003). Applied Longitudinal Data Analysis for Epidemiology - A Practical Guide. NY: Cambridge University Press.

Vries S de, (2011) Authorised Dutch translation of the IMR-scales, Hogeschool Saxion Deventer.

Wing, J., Beevor, A., Curtis, R., Park, S., Hadden, S. & Burns, A. (1998) Health of the Nation Outcome Scales (HoNOS): Research and development. British Journal of Psychiatry, 172, 11-18.

Young, S. & Bullock, W. (2003) Illness Management and Recovery and the Role of the Mental Health Recovery Measure (MHRM) in Outcomes Research.Ohio Department of Mental Health,Ohio Coordinating Center for Excellence for Illness and Recovery.

Zimet G.D., Powell S.S., Farley G.K. et al. (1990), Psychometric Characteristics of the Multidimensional Scale of Perceived Social Support, Journal of Personality Assessment 55: 610-617.

Zubin J, Spring B. Vulnerability: a new view of schizophrenia. J Abnorm Psychol. 1977;86:103–126.

*Guidelines and Implementation sets (Richtlijnen en implementatiesets):*

Multidisciplinaire richtlijn schizofrenie voor de diagnostiek, behandeling en zorgorganisatie van volwassen cliënten met schizofrenie, kwaliteitsinstituut voor de gezondheidszorg CO, Utrecht, 2003 (Multidisciplinary guideline for the diagnosis, treatment and care organization of adult patiënts with schizophrenia)

Multidisciplinaire richtlijn schizofrenie 2005 richtlijn voor de diagnostiek, zorgorganisatie en behandeling van volwassenen cliënten met schizofrenie onder auspiciën van de landelijke stuurgroep multidisciplinaire richtlijnontwikkeling in de ggz. (2005 guideline for multidisciplinary schizophrenia diagnosis, care organization and treatment of adult patiënts with schizophrenia under the auspices of the National Steering Committee multidisciplinary development of mental health care).

Samsha (2002). Illness Management & recovery implementation resource kit, draft version, IMR workbook, 2002, US Department of health and human services, substance abuse and mental health services administration, centre for mental health services.

## Annex 1 Flow chart randomisation of participants

**Expected number of patients required for inclusion in the study (using drop-out ratio’s of the pilot-study)**

**(At randomization assignment to experimental and control group in a ratio of 3:2)**

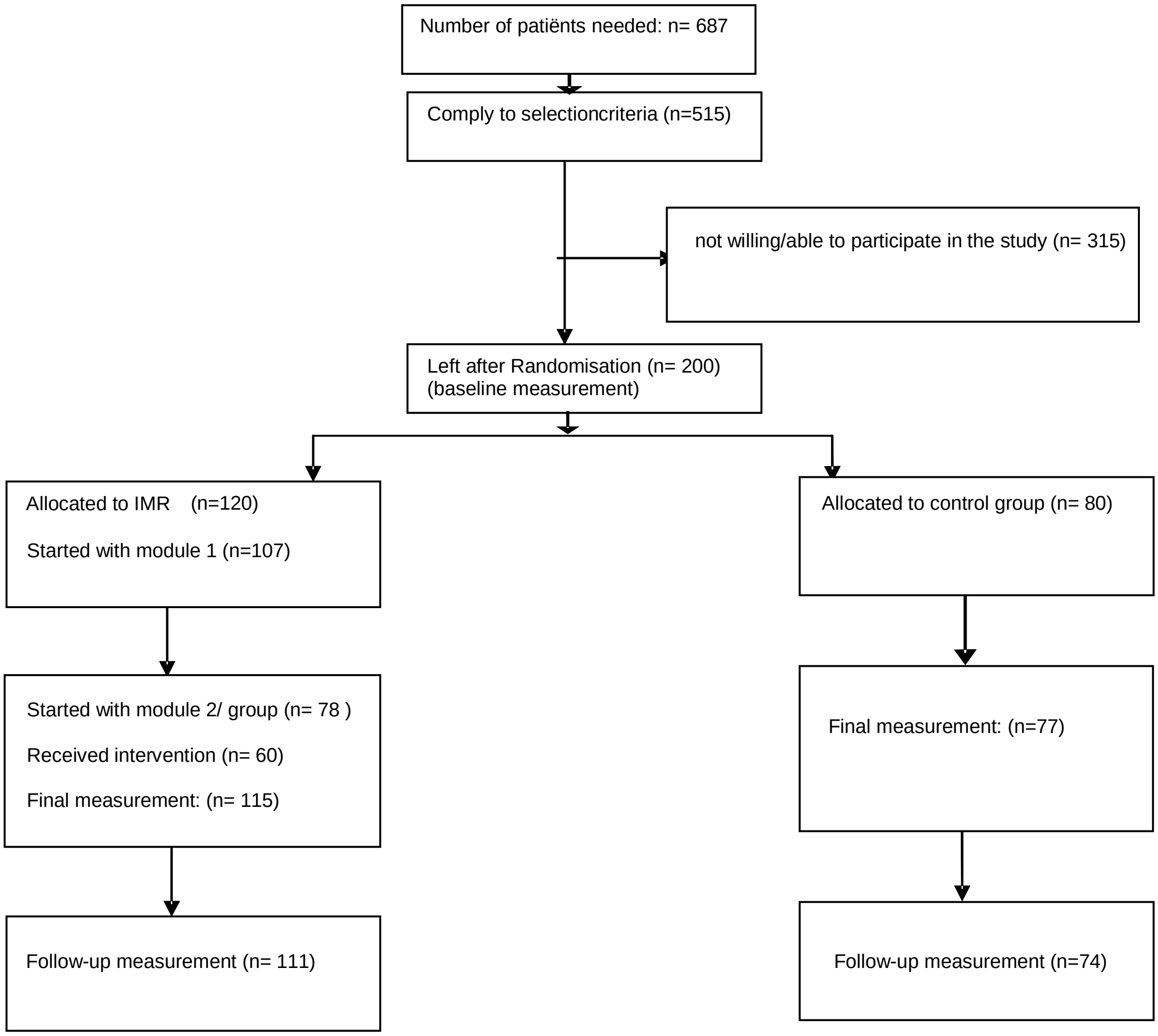

**Annex 2 Budget Trial Illness Management and Recovery (IMR) at Bavo Europoort**

**Total budget of the study: € 337.830,-**

- 0.8 FTE academic researcher for five years (**250,000 Euro**)

The researcher is funded by the Parnassia Bavo Group
(largely by Bavo Europoort and a smaller part by the Parnassia Bavo Academy).

- (3 x 167 =) 501 interviews:

| | |
|---|---|
| - Independent interviewers à € 30,- per interview = 660 x € 30,- = | € 19.800, - |
| - For patients: 3 interviews (€ 20 + € 25 + € 30 =) € 75 per patient x 200 = | € 15.000,-. |
| **Total for the interviews:** | **€ 34.800,-** |

- Research assistant 2 days per week: about 1000 euros gross per month (scale 45 / 6) including employer's costs: for 3 years: **€ 42.530,-**

- Other costs: **€ 10.500**,-
o Informationbrochures & posters on the research (€ 2000,-)
o Costs of photocopying of the questionnaires (€ 500,-)
o Traveling expenses of researcher and interviewers € 4000,-
o Visiting 2 international conferences (2 x € 2000,- =) € 4000,-

- Advise by supervisory committee: PM

**Annex 3 Overview Instruments + research contacts per patient**

| | Source of information | | | | Time | Moment of Measurement | | |
|---|---|---|---|---|---|---|---|---|
| | dossier | patient | clinician | instrument | | M1 | M2 | M3 |
| | | | | | | | | |
| Demographic data | **x** | | | | | **x** | | |
| Diagnosis | **x** | | | | | **x** | | |
| Care Consumption | **x** | | | | | **x** | **x** | **x** |
| Goals | | **x** | | Granholm's Goals Template | 15 min | **x** | **x** | **x** |
| Illness management | | **x** | | IMR-scale-patient version | 10 min | **x** | **x** | **x** |
| Illness management | | | **x** | IMR-scale clinician version | | **x** | **x** | **x** |
| Coping | | **x** | | CSES | 10 min | **x** | **x** | **x** |
| Social Support | | **x** | | MSPSS | 8 min | **x** | **x** | **x** |
| Treatment compliance | | | **x** | SES | | **x** | **x** | **x** |
| Insight | | **x** | | Insight Scale (IS) | 7 min | **x** | **x** | **x** |
| Symptoms | | **x** | | BSI | 10 min | **x** | **x** | **x** |
| Addiction | | **x** | | item 24 of the ASI | 5 min | **x** | **x** | **x** |
| Recovery | | **x** | | MHRM | 10 min | **x** | **x** | **x** |
| Self- Stigma | | **x** | | Ismi | 10 min | **x** | **x** | **x** |
| Self-Esteem | | **x** | | SERS-SF | 7 min | **x** | **x** | **x** |
| Quality of Life | | **x** | | The EQ-5D | 7 min | **x** | **x** | **x** |
| Satisfaction | | **x** | | Two questions of the ROM | 6 min | **x** | **x** | **x** |
| Social Functioning | | **x** | | The SF Scale | 15 min | **x** | **x** | **x** |
| | | | | | tot.min. | 120 | 120 | 120 |

**Annex 4: Flowchart researchprotocol Trial Illness Management and Recovery (IMR) in Bavo Europoort**

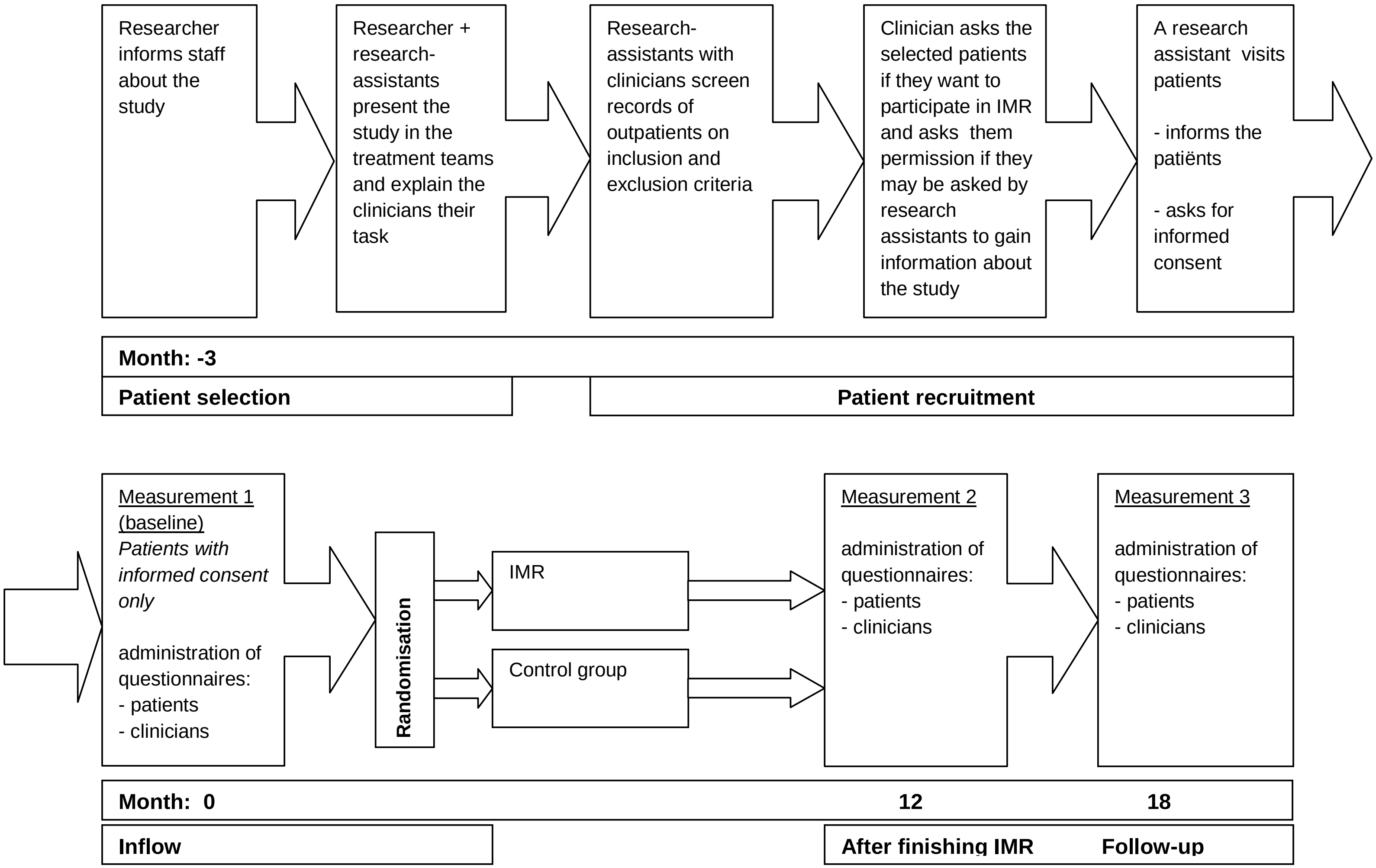

Supplement: Supplementary Data Sheet 1 — Positive decision Erasmus MC. [file Data_Sheet_1.PDF]
