# Supplementary material for: Effects of Illness Management and Recovery: A Multicenter Randomized Controlled Trial
Source: Front Psychiatry. 2021 Dec 14;12:723435. doi: 10.3389/fpsyt.2021.723435 (PMC8712643; doi:10.3389/fpsyt.2021.723435)
Supplement: Supplementary Data Sheet 2 — Original study Protocol for the METC. [file Data_Sheet_2.PDF]

Erasmus MC
Universitair Medisch Centrum Rotterdam

Medische Ethische Toetsings Commissie
Erasmus MC
www.erasmusmc.nl/commissies/metc/

Drs. B.J. Roosenschoon
Parnassia Bavo Groep
Monsterseweg 83
2553 RJ Den Haag

Doorkiesnummer +31 10 7033625/34428
Faxnummer
Kamernummer Fd 209
E-mail metc@erasmusmc.nl
Ons kenmerk MdV/ss/METC159081
Datum 27 augustus 2012

Betreft: MEC-2012-142 Positief besluit NL38605.078.12 v02

Postadres
Postbus 2040
3000 CA Rotterdam

Bezoekadres
Dr. Molewaterplein 50
3015 GE Rotterdam

Titel onderzoek:
'Trial Illness Management and Recovery (IMR) in Bavo Europoort'

Verrichter: Parnassia Bavo Groep
Singlecenter

Geachte heer Roosenschoon,

De Medisch Ethische Toetsings Commissie Erasmus MC (METC) heeft het bovenvermelde onderzoeksvoorstel ontvangen op 9 maart 2012.

Besluit
De commissie heeft het onderzoeksvoorstel in het kader van de Wet medisch-wetenschappelijk onderzoek met mensen (WMO) besproken in de 865^{e} vergadering d.d. 24 april 2012. Naar aanleiding van deze toetsing heeft de commissie u enkele vragen c.q. opmerkingen voorgelegd. De vragen en opmerkingen zijn naar tevredenheid beantwoord.

De commissie oordeelt **positief** over de uitvoering van het onderzoek in het volgende centrum:

- Parnassia Bavo Groep te Den Haag

Alvorens met de uitvoering van het onderzoek in het hierboven vermelde centrum gestart mag worden, dient u in het bezit te zijn van toestemming voor de uitvoering van het onderzoek door de Raad van Bestuur / Directie van het betreffende centrum. De METC Erasmus MC ontvangt graag een kopie van de toestemming voor de uitvoering.

WMO proefpersonenverzekering
Het onderzoek valt onder de proefpersonenverzekering van de verrichter (Parnassia Bavo Groep).

Aansprakelijkheidsverzekering
De commissie heeft verder vastgesteld dat door de verrichter (Parnassia Bavo Groep) is voorzien in de dekking van een aansprakelijkheidsverzekering.

Voorzitters
Prof.dr. H.W. Tilanus
Prof.dr. H.J. Metselaar

Vice voorzitter
Dr. C.M. Zwaan

Secretarissen
Mw. mr. C.P. Bron-van Vliet
Mw. drs. A.I.J.M. Schellevis-Mintiens
Mw.dr.ir. M.M.C. de Vries-Velraeds
Mw.ing. W.C.M. Tielemans

Secretaressen
Mw. G.J. Slag
Mw. S. Sneevliet
Mw. H.L. Lammers-Assems
Mw. A. de Jong

Adm. medewerker
Mw. A.E. Huukskoot

Het secretariaat is geopend van maandag tot en met vrijdag van 08.30 tot 17.00 uur

Erasmus MC
Universitair Medisch Centrum Rotterdam

Melden
De volgende gegevens moeten aan de commissie worden gemeld:

- Startdatum (datum waarop de eerste proefpersoon is geïncludeerd)
- Einde inclusie (datum waarop de laatste proefpersoon is geïncludeerd)
- Einddatum (datum waarop de laatste meting bij de laatste proefpersoon heeft plaatsgevonden)

Voorleggen ter beoordeling
De volgende documenten moeten aan de commissie ter beoordeling worden voorgelegd:

- Onverwachte en ernstige bijwerkingen via portal ToetsingOnline
- Amendementen en/of addenda
- Formulier Jaarlijkse voortgangsrapportage, voor het eerst één jaar na dit besluit
- Publicaties en eindrapport

BROK cursus
Voorts wijst de commissie de onderzoekers erop dat, conform landelijke afspraken, klinisch onderzoekers verplicht zijn de "Basiscursus regelgeving en organisatie van klinisch onderzoek" (BROK) te doorlopen en het bijbehorende certificaat te behalen. De commissie gaat ervan uit dat, indien dit nu nog niet het geval is, de klinisch onderzoekers van deze studie maximaal 6 maanden na aanvang van de studie aan deze verplichting voldaan hebben. Voor informatie over de inhoud van de BROK-cursus kunt u terecht bij dr. R.E. Juttmann, Directie O&O, intern tel.nr. 32192. Voor praktische informatie (zoals data waarop de cursus plaatsvindt en inschrijving) kunt u contact opnemen met het Congresbureau, intern tel.nr. 31621.

Beroep
Tot slot wijs ik u erop, dat op grond van artikel 23 van de Wet medisch-wetenschappelijk onderzoek met mensen juncto artikel 7: 1 van de Algemene wet bestuursrecht, degene wiens belang rechtstreeks bij dit besluit is betrokken daartegen binnen zes weken na de dag waarop dit besluit bekend is gemaakt, een administratief beroepschrift kan indienen bij de Centrale Commissie Mensgebonden Onderzoek. Een dergelijk administratief beroepschrift dient u te adresseren aan: CCMO, Postbus 16302, 2500 BH Den Haag.

Geldigheid besluit
De commissie heeft de bevoegdheid haar positieve besluit in te trekken als vaststaat dat de uitvoering van het onderzoek ernstig tekortschiet. Het positieve besluit van de commissie verliest geldigheid als met de uitvoering van het onderzoek niet is begonnen binnen één jaar nadat dit besluit is genomen.

METC fee
Het onderzoeksproject wordt door de industrie ondersteund. Derhalve wordt een tegemoetkoming in de kosten van het beoordelingstraject door de commissie (METC-fee) in rekening gebracht. De METC-fee voor een singlecenter WMO besluit bedraagt € 2.000.

Met vriendelijke groet,
namens de Medisch Ethische Toetsings Commissie Erasmus MC,

Mw.dr.ir. M.M.C. de Vries-Velraeds
Secretaris

Erasmus MC
Universitair Medisch Centrum Rotterdam

Beoordeelde documenten

- Aanbiedingsbrief en akkoordverklaring coördinerend onderzoeker prof.dr. C.L. Mulder, afdelingshoofd prof.dr. W.J.J. Hoogendijk en uitvoerend hoofdonderzoeker drs. B.J. Roosenschoon d.d. 5 maart 2012;
- ABR-formulier nr. NL38605.078.12, versie 02 d.d. 12 juli 2012;
- Addendum bij ABR-formulier, zoals ontvangen d.d. 9 maart 2012;
- Onderzoeksprotocol, versie 2 d.d. 5 juli 2012;
- Patiënteninformatie- en toestemmingsformulier Bavo Europoort, versie 3, 21 augustus 2012;
- Folder Hersteltraining, dec.'11/113;
- Diverse vragenlijsten, zoals ontvangen d.d. 9 maart 2012:
    - Illness Management and Recovery Scale: IMR schaal; cliënten versie,
    - Illness Management and Recovery Scale: IMR schaal, hulpverleners versie,
    - Coping Self-Efficacy Scale;
    - Multidimensional Scale of Perceived Social Support;
    - Schaal voor Therapietrouw;
    - Psychose Inzicht Schaal;
    - Korte psychiatrische beoordelingsschaal (BRPS-E);
    - The Addiction Severity Index Item 24;
    - Mental Health Recovery Measure – Nederlandse versie;
    - UCSD/VASDHS Schizophrenia Psychosocial Rehabilitation Program Granholm's Goals Template;
    - Vragenlijst Geïnternaliseerd Stigma van Pschychische aandoeningen;
    - Zelfwaarderingslijst;
    - Euro-Qol 5D;
    - Euro-Qol gezondheidstoestand;
    - Vragenlijst tevredenheid;
    - Sociaal functioneren;
- Brief Symptom Inventory, zoals ontvangen d.d. 16 juli 2012;
- Verzekeringscertificaat d.d. januari 2012 inzake WMO-proefpersonenverzekering verrichter (Stichting Parnassia Bavo Groep);
- Verzekeringscertificaat d.d. januari 2012 inzake Aansprakelijkheidsverzekering verrichter (Stichting Parnassia Bavo Groep);
- Curriculum vitae onafhankelijke arts, drs. S. Wolters, getekend d.d. 20 februari 2012;
- Curriculum vitae coördinerend onderzoeker, prof.dr. C.L. Mulder, getekend d.d. 13 februari 2012;
- Curriculum vitae hoofdonderzoeker, drs. B.J. Roosenschoon, getekend d.d. 22 februari 2012;
- Informatie deelnemend centrum, zoals ontvangen d.d. 9 maart 2012;
- Onderzoeksverklaring Bavo Europoort d.d. 23 februari 2012;
- Overeenkomst Educational Grant Algemeen tussen Janssen-Cilag B.V. en Bavo Europoort, getekend 1, 5 en 21 maart 2012; en
- Brief van Janssen-Cilag B.V. inzake financiële ondersteuning d.d. 20 december 2012.

Erasmus MC
Universitair Medisch Centrum Rotterdam

Samenstelling METC Erasmus MC

Dr. D.A.M.P.J. Gommers	Anesthesioloog
Dr.ir. M.T. Hilhorst	Ethicus
Mw.dr. B.C.P. Koch	Ziekenhuisapotheker / Klinisch farmacoloog
Mw.dr. E. van Meerten	Internist-oncoloog
Prof.dr. H.J. Metselaar (voorzitter)	Internist / MDL-arts
Prof.dr. E.W. Steyerberg	Methodoloog
Mw.mr. C.F.B. Verhagen-van Weerden	Gezondheidsjurist
Mw.dr.ir. M.M.C. de Vries-Velraeds	Secretaris
Mw.drs. S.E. Zijlstra	Invalshoek proefpersoon / Extern lid

Cc. Prof.dr. C.L. Mulder
CCMO (digitaal verzenden)
